# Dentate Gyrus Engrams in Fear and Reward: Mechanistic Principles, Critical Gaps, and Paths to Translation

**DOI:** 10.1111/jnc.70413

**Published:** 2026-03-19

**Authors:** Lorianna M. Colón, Oluwatoni A. Famuyide, Amelia J. Eisch

**Affiliations:** ^1^ Division of Basic Science, Department of Anesthesiology and Critical Care Children's Hospital of Philadelphia (CHOP) Philadelphia Pennsylvania USA; ^2^ Cornell University, College of Arts and Sciences Ithaca New York USA; ^3^ Neuroscience Graduate Group, Biomedical Graduate Studies University of Pennsylvania Perelman School of Medicine (PSoM) Philadelphia Pennsylvania USA; ^4^ Department of Anesthesiology and Critical Care PSoM Philadelphia Pennsylvania USA; ^5^ Department of Neuroscience PSoM Philadelphia Pennsylvania USA

**Keywords:** dentate gyrus, engram, fear, memory, reward

## Abstract

The hippocampal dentate gyrus (DG) has emerged as a cornerstone of engram research. While DG fear‐based engrams have been extensively studied, revealing principles of allocation, consolidation, retrieval, and valence switching, engrams encoding context‐reward associations, particularly those involving drugs of abuse, remain comparatively underexplored. This knowledge gap has critical implications for understanding addiction, depression, and other disorders involving dysfunctional reward processing. In this review, we first establish the DG's unique anatomical and functional properties that position it as an ideal model system for engram research. We then systematically examine the DG fear engram literature, documenting how decades of contextual fear conditioning studies have elucidated mechanisms of competitive allocation, molecular consolidation, competing extinction ensembles, and context‐dependent discrimination versus generalization. Turning to reward engrams, we synthesize emerging evidence demonstrating that drug‐context associations are encoded through sparse, distributed ensembles across multiple brain regions including the nucleus accumbens, prefrontal cortex, amygdala, and hippocampus. While these studies establish foundational principles of reward engram allocation and retrieval, critical mechanistic gaps remain, particularly regarding differences between drug‐associated and natural reward memories, the neural coding of context versus valence in hippocampal circuits, and the mechanisms underlying drug associated reward memory extinction. Evidence from valence switching studies demonstrates that the DG processes and stores both fear and reward memories within overlapping circuits, exhibiting remarkable plasticity in linking contextual representations to opposing emotional outcomes, a flexibility distinguishing it from structures with hardwired valence encoding. This encoding capacity positions the DG as a promising target for interventions aimed at modifying pathological emotional associations in addiction and trauma‐related disorders while preserving contextual specificity. Understanding reward engram mechanisms with the same rigor applied to fear engrams is essential for developing comprehensive frameworks of how DG circuits contribute to memory‐related psychopathology and for translating engram research into therapeutic applications.

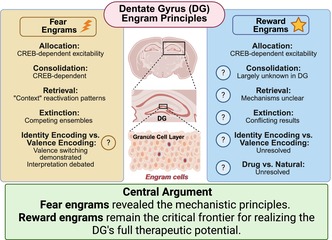

## Graphical abstract

Memories of rewarding experiences powerfully shape behavior, yet when maladaptive, they can drive psychiatric conditions including addiction, depression, and PTSD. Memory traces, or engrams, have been extensively studied for fear memories in the hippocampal dentate gyrus. However, engrams for rewarding experiences remain comparatively underexplored, a significant gap given that many psychiatric conditions involve disrupted reward processing. This review compares fear and reward engram research, identifying critical knowledge gaps. Importantly, the dentate gyrus can flexibly reassociate contexts with different emotional outcomes, unlike brain regions with fixed emotional coding. This plasticity positions it as a promising therapeutic target for memory‐related psychiatric disorders.

**Abbreviations**
BLA: basolateral amygdala
CA1: cornu Ammonis 1
CA2: cornu Ammonis 2
CA3: cornu Ammonis 3
CFC: contextual fear conditioning
ChR2: channelrhodopsin‐2
CNS: central nervous system
CPP: conditioned place preference
d: dorsal
DG: dentate gyrus
DH: dorsal hippocampus
DMS: dorsomedial striatum
EC: entorhinal cortex
ELS: early‐life stress
GC: granule cell
IEG: immediate‐early genes
LC: locus coeruleus
LEC: lateral entorhinal cortex
MEC: medial entorhinal cortex
MIA: immune activation
NAc: nucleus accumbens
OFC: orbitofrontal cortex
PFC: prefrontal cortex
PTSD: post‐traumatic stress dcisorder
SST+: somatostatin‐positive
V: ventral

## 1 Introduction

When memory systems falter, the consequences are profound. Memory dysfunction underlies some of our most challenging psychiatric disorders. Traumatic flashbacks trap patients in cycles of fear and avoidance, while addiction hijacks reward systems to prioritize drug‐seeking over adaptive behaviors. Depression often involves both excessive fear sensitivity and blunted reward processing. These conditions arise, at least in part, from pathological memory processing that could potentially be addressed through targeted therapeutic intervention.

Over a hundred years ago, Richard Semon introduced the term *engram* to describe the putative physical substrate of memory (Semon 1921). In its modern usage, the engram refers to the enduring cellular and molecular changes within discrete neuronal populations that arise from learning and allow later recall (Josselyn and Tonegawa 2020). Identifying an engram requires demonstration of functional relevance; neuronal ensembles must be shown to causally drive memory‐related behavior through manipulation experiments. Specifically, inhibiting these ensembles should impair memory expression, while artificially reactivating them should be sufficient to induce memory recall. Without such functional validation, active neuronal populations represent ensembles but cannot be definitively classified as engrams. This functional criterion distinguishes correlative observations of memory‐associated activity from causal engram mechanisms. Engrams meeting these criteria have been identified in multiple brain regions and for different types of memory (see reviews Tonegawa, Liu, et al. 2015; Bostancıklıoğlu 2020).

The trajectory of engram research has been shaped by both technical innovation and conceptual frameworks around regional specialization. The amygdala provided early proof‐of‐concept for engram manipulation, with seminal work showing that neurons could be genetically recruited into an engram, tagged, and then selectively silenced (Han et al. 2007, 2009; Reijmers et al. 2007). These findings confirmed that engrams were not merely theoretical constructs but could be localized and experimentally controlled. Building on this foundation, the hippocampal dentate gyrus (DG) emerged as a model system for engram research due to its sparse coding properties, pattern separation capabilities, and experimental accessibility. The hippocampus had been characterized as essential for spatial and contextual memory as well as cognitive functions critical for navigating environments and distinguishing experiences. This functional profile made contextual fear conditioning (CFC) an especially attractive behavioral paradigm: it combines spatial and contextual processing with reliable behavioral readouts and precise temporal structure. The synergy between the DG's established role in contextual processing and the methodological advantages of fear conditioning established CFC as the predominant approach for hippocampal engram research (see Table 1).

**TABLE 1:** Dentate Gyrus (DG) engram research presented by theme.

Theme	Behavior paradigm(s) used	Engram manipulation	References	Summary
Section 2.3 Adult neurogenesis and memory				
	Contextual fear conditioning	Optogenetic inhibition	Denny et al. 2014	Optogenetic inhibition of DG engram cells during recall attenuated fear expression, demonstrating their necessity for memory retrieval
	Contextual fear conditioning	Optogenetic stimulation	Guskjolen et al. 2018	Optogenetic DG stimulation recovers forgotten infant memories in adult mice
	Contextual fear conditioning	Optogenetic stimulation	Golbabaei et al. 2025	Adult neurogenesis progressively degrades DG engram specificity, promoting generalization across contexts
	Contextual fear conditioning, Water maze	Optogenetic inhibition/stimulation	Ko et al. 2025	Neurogenesis‐dependent reorganization of DG‐CA3‐CA1 engram circuitry drives time‐dependent shifts from precise episodic to generalized gist memories; eliminating neurogenesis preserves memory precision while promoting neurogenesis accelerates generalization
Section 3.1 Foundational discoveries				
	Contextual fear conditioning	Optogenetic stimulation	Liu et al. 2012	Reactivating tagged fear‐encoding DG neurons was sufficient to trigger memory recall
	Contextual fear conditioning	Optogenetic stimulation	Ramirez et al. 2013	Optogenetic reactivation of memory engram–bearing cells in the DG, but not CA1, induced false memories
Section 3.2 Engram allocation and competitive dynamics				
	Contextual fear conditioning	Chemogenetic inhibition, Optogenetic inhibition	Park et al. 2016	Engram allocation parallels in LA and DG; CREB+ DG neurons drive fear memory
	Contextual fear conditioning, Elevated plus maze, Spatial navigation task	Chemogenetic stimulation, Optogenetic inhibition	Stefanelli et al. 2016	Lateral inhibition by SST+ neurons controls DG engram size and fear memory stability
	Contextual fear conditioning	Optogenetic stimulation	Pignatelli et al. 2019	Kir2.1 channel controls engram cell excitability
	Contextual fear conditioning, Barnes maze, Enriched environment	Optical stimulation	Dovek et al. 2025	Highly excitable semilunar GCs are more likely to be recruited to engrams
Section 3.3 Memory consolidation and silent engrams				
	Contextual fear conditioning	Protein synthesis inhibition; Optogenetic stimulation	Ryan et al. 2015	Disrupting consolidation with anisomycin blocks fear memory but can be restored with DG engram stimulation
	Contextual fear conditioning	Chemogenetic inhibition, Optogenetic inhibition and stimulation	Tomé et al. 2024	Inhibitory neuron activity during consolidation is critical for engram stability and memory specificity
	Contextual fear conditioning	Optogenetic inhibition and stimulation	Kitamura et al. 2017	DG engram cells support the maturation of PFC engram cells
	Contextual fear conditioning	Optogenetic stimulation	Roy et al. 2017	Disrupting memory recall with anisomycin blocks retrieval but can be restored with DG engram stimulation
	Contextual fear conditioning	Chemogenetic inhibition	Rao‐Ruiz, Yu, et al. 2019	The sustained expression of Arc, driven by CREB during consolidation, is essential for DG engram stability
	Contextual fear conditioning	Optogenetic stimulation	Guskjolen et al. 2018	Optogenetic reactivation of DG engrams recovers inaccessible infant memories in adults.
	Object‐location recognition	Optogenetic stimulation	Bolsius et al. 2023	Optogenetic DG engram reactivation or Roflumilast treatment can recover object‐location memories during sleep deprivation
Section 3.4 Memory retrieval and reactivation dynamics				
	Contextual fear conditioning	Optogenetic inhibition	Denny et al. 2014	Optogenetic inhibition of DG engram cells during recall attenuated fear expression, demonstrating their necessity for memory retrieval
	Contextual fear conditioning, Place preference/avoidance	Optogenetic stimulation	Redondo et al. 2014	Optogenetic reactivation of DG engrams during conditioning with opposite valence switched emotional association from fear to reward (or vice versa), demonstrating plasticity in valence encoding
	Contextual fear conditioning	Optogenetic stimulation	Ryan et al. 2015	Memory retention associated with multiple interconnected engram ensembles
	Contextual fear conditioning	Optogenetic stimulation	Roy et al. 2017	Disrupting memory recall with anisomycin blocks retrieval but can be restored with DG engram stimulation
	Contextual fear conditioning	Optogenetic stimulation	Guskjolen et al. 2018	Optogenetic DG stimulation recovers forgotten infant memories in adult mice
	Contextual fear conditioning	Chemogenetic stimulation	Ressler et al. 2021	Backward conditioning indirectly retrieved hippocampal contextual fear engrams; chemogenetic capture and reactivation of hippocampal ensembles was sufficient to drive fear, and post‐retrieval protein synthesis inhibition attenuated the indirectly retrieved memory, demonstrating reconsolidation sensitivity
	Contextual fear conditioning	Chemogenetic inhibition	Khalaf et al. 2018	Continued DG engram activity is critical for remote fear
	Contextual fear conditioning	Optogenetic stimulation	Smith et al. 2018	Dietary polyphenols boost fear memory recall by increasing neuron recruitment to the DG engram
	Contextual fear conditioning	Optogenetic inhibition and stimulation	Lacagnina et al. 2019	Retrieval of fear and retrieval of extinction recruit unique DG ensembles
	Contextual fear conditioning	Optogenetic stimulation	Dorst et al. 2024	Engram reactivation drives context‐dependent freezing and distinct network activity
	Contextual fear conditioning, Forced swim test, Novel object recognition, Y maze	Optogenetic stimulation	Perusini et al. 2017	Optogenetic stimulation of DG engram can rescue Alzheimer's memory deficits
	Contextual fear conditioning	Optogenetic stimulation	Suthard et al. 2024	Optogenetic engram recall mimics natural neuronal‐astrocyte fear dynamics
	Contextual fear conditioning	Chemogenetic stimulation	Jin et al. 2025	Downregulated NPTX (cell adhesion molecule) destabilizes DG engram; rescued by pharmacological or chemogenetic activation of DG engram
Section 3.5 Fear memory processing: Competing ensembles and extinction dynamics				
	Contextual fear conditioning	Optogenetic inhibition and stimulation	Lacagnina et al. 2019	Unique DG ensembles for fear/extinction manage fear expression
	Contextual fear conditioning	Chemogenetic inhibition, Chemogenetic stimulation	Gong et al. 2022	Activating dDG engrams during extinction enhances extinction and prevents renewal
	Contextual fear conditioning	Chemogenetic inhibition	Khalaf et al. 2018	Original fear engram actively contributes to remote fear extinction
	Contextual fear conditioning	Optogenetic inhibition, and stimulation	Kitamura et al. 2017	DG engram cells support the maturation of PFC engram cells
Section 3.6 Contextual specificity: Mechanisms of discrimination versus generalization				
	Contextual fear conditioning, Novel environment	Optogenetic inhibition	Bernier et al. 2017	Inhibition of DG engrams impacts fear acquisition, discrimination, and generalization
	Contextual fear conditioning, Open field, Elevated plus maze, Light/Dark test, Tail suspension	Optogenetic inhibition and stimulation	Guo et al.2018	A cytoskeletal protein in mossy fiber terminals regulates DG GC connectivity with CA3 INs controlling remote memory precision/generalization
	Spatial navigation tasks	Optogenetic inhibition and stimulation	Hainmueller and Bartos 2018	Adult‐born dentate granule cells integrate into spatial memory circuits and contribute to the encoding of novel spatial environments, demonstrating their unique role in pattern separation during navigation
	Contextual fear conditioning	Optogenetic inhibition and stimulation	Lacagnina et al. 2019	Retrieval of fear and retrieval of extinction recruit unique DG ensembles
	Contextual fear conditioning	Chemogenetic inhibition and stimulation, Optogenetic inhibition and stimulation	Sun et al. 2020	Fos‐ and Npas4‐defined ensembles reflect DG functional heterogeneity, balancing memory generalization and discrimination via distinct synaptic mechanisms
	Contextual fear conditioning	Chemogenetic inhibition and stimulation, Optogenetic inhibition and stimulation	Cui et al. 2024	Reactivating a fear engram in a safe environment triggers memory updating/reduced generalization
	Contextual fear conditioning, Spatial navigation tasks	Optogenetic inhibition and stimulation	Kheirbek et al. 2013	Optogenetic manipulation of dentate gyrus activity revealed that increasing DG activity enhances pattern separation and reduces anxiety‐like behavior, while decreasing activity impairs discrimination between similar contexts
	Contextual fear conditioning	Chemogenetic inhibition	Lesuis et al. 2021	Chemogenetic suppression of DG engram reduced corticosterone ‐induced fear generalization
	Contextual fear conditioning, Open field, Elevated plus maze	Electrophysiological inhibition/stimulation	Lin et al. 2025	Excitability and autophagy in dDG engram are the cellular basis of fear generalization
Section 3.7 Stress and early life effects				
	Contextual fear conditioning	Optogenetic stimulation	Finkelstein et al. 2022	Social stress reactivates previous DG fear engrams driving fear behavior through memory coalescence
	Chronic immobilization stress, Open field, Sucrose preference, Object‐female association, Elevated plus maze, Novelty‐suppressed feeding, Tail suspension	Optogenetic stimulation	Ramirez et al. 2015	Activating positive memory DG engrams can suppress stress induced depression‐like behavior
	Contextual fear conditioning, novel object recognition, barnes maze, three chamber social interaction	Optogenetic stimulation	Power et al. 2023	Infantile amnesia is a reversible retrieval deficit determined by maternal immune state
Section 3.8 Circuit level and network interactions				
	Contextual fear conditioning, Elevated plus maze, Spatial navigation task	Chemogenetic stimulation, Optogenetic inhibition	Stefanelli et al. 2016	SST‐GC microcircuit; Lateral inhibition by SST+ neurons controls DG engram size and fear memory stability
	Contextual fear conditioning	Optogenetic inhibition and stimulation	Kitamura et al. 2017	DG engram cells support the maturation of PFC engram cells
	Contextual fear conditioning, Open field, Elevated plus maze, Light/Dark test, Tail suspension	Optogenetic inhibition and stimulation	Guo et al. 2018	A cytoskeletal protein in mossy fiber terminals regulates DG GC connectivity with CA3 INs controlling remote memory precision/generalization
	Contextual fear conditioning	Chemogenetic inhibition, Optogenetic stimulation	Dai et al. 2023	LC‐DG circuit promotes extinction by expanding DG engram size
	Contextual fear conditioning	Chemogenetic inhibition and stimulation Optogenetic stimulation	Roy et al. 2022	Memory engrams for a contextual fear conditioning memory are distributed across multiple brain regions
	Contextual fear conditioning	Optogenetic stimulation	Dorst et al. 2024	Engram reactivation selectively engages different network hub regions based on context
Section 4.4 Context identity, valence encoding, and memory plasticity				
Fear and Reward	Contextual fear conditioning, Place preference/avoidance	Optogenetic stimulation	Redondo et al. 2014	Optogenetic reactivation of DG engrams during conditioning with opposite valence switched emotional association from fear to reward (or vice versa), demonstrating plasticity in valence encoding
Fear and Reward	Chronic immobilization stress, Open field, Sucrose preference, Object‐female association, Elevated plus maze, Novelty‐suppressed feeding, Tail suspension	Optogenetic stimulation	Ramirez et al. 2015	Activating positive memory DG engrams can suppress stress induced depression‐like behavior
Fear and Reward	Contextual fear conditioning, Place preference/avoidance, Open field, Female exposure	Optogenetic stimulation	Chen et al. 2019	Reactivating tagged dorsal/ventral DG engrams drives freezing, avoidance, or place preference depending on the original memory's valence
Fear and Reward	Open field, Novel environment, Radial arm maze, Restraint stress, Contextual fear conditioning, Female exposure	Optogenetic stimulation	Grella et al. 2022	Optogenetically reactivating positive memory engrams (female exposure, VTA self‐stimulation, or cocaine) in the dorsal dentate gyrus during fear memory reconsolidation successfully disrupts conditioned fear in mice, while reactivating negative (restraint stress, fear conditioning) or neutral (home cage) memory engrams is ineffective
Reward	Morphine injections, Place aversion	Chemogenetic inhibition, Optogenetic stimulation and inhibition	Dai et al. 2023	Optogenetic activation of the LC‐DG circuit before recall‐extinction promotes extinction of opioid withdrawal memory by expanding the population of recall‐tagged DG engram cells reactivated during extinction, demonstrating that LC input modulates memory updating through enlarging the active engram ensemble.
Fear and Reward	Contextual fear conditioning, Place preference, Morris water maze	Chemogenetic stimulation	Cai et al. 2025	Repeated oxytocin administration into the DG prevents retrieval of methamphetamine‐associated reward memories, spatial memories, and contextual fear memories by enhancing adult hippocampal neurogenesis.
Section 5 Therapeutic implications				
	Contextual fear conditioning, Spatial navigation tasks	Optogenetic inhibition and stimulation	Kheirbek et al. 2013	Optogenetic manipulation of dentate gyrus activity revealed that increasing DG activity enhances pattern separation and reduces anxiety‐like behavior, while decreasing activity impairs
	Contextual fear conditioning	Optogenetic stimulation	Roy et al. 2017	Disrupting memory recall with anisomycin blocks retrieval but can be restored with DG engram stimulation
	Contextual fear conditioning, forced swim test, novel object recognition, Y maze	Optogenetic stimulation	Perusini et al. 2017	Optogenetic stimulation of DG engram can rescue Alzheimer's memory deficits
	Contextual fear conditioning	Optogenetic stimulation	Smith et al. 2018	Dietary polyphenols boost fear memory recall by increasing neuron recruitment to the DG engram
	Contextual fear conditioning	Chemogenetic stimulation	Ressler et al. 2021	Backward conditioning indirectly retrieved hippocampal contextual fear engrams; chemogenetic capture and reactivation of hippocampal ensembles was sufficient to drive fear, and post‐retrieval protein synthesis inhibition attenuated the indirectly retrieved memory, demonstrating reconsolidation sensitivity
	Contextual fear conditioning	Chemogenetic stimulation	Jin et al. 2025	Downregulated NPTX (cell adhesion molecule) destabilizes DG engram; rescued by pharmacological or chemogenetic activation of DG engram

*Note:* Summary of studies investigating DG memory engrams organized by research themes which correspond to sections of this review. Studies were identified through PubMed searches (through January 2026) using combinations of keywords including “dentate gyrus,” “engram,” “memory ensemble,” “contextual fear conditioning,” “reward memory,” and “hippocampus,” supplemented by manual screening of reference lists from identified papers and recent reviews. Inclusion criteria required: (1) functional manipulation of identified neuronal ensembles (optogenetic, chemogenetic, or pharmacological), (2) demonstration that manipulated ensembles were allocated during specific learning experiences, and (3) assessment of causal contributions to memory‐related behavior through necessity (inhibition impairs memory) or sufficiency (activation drives memory) experiments. This table focuses on studies directly manipulating DG engram populations and is not exhaustive of all hippocampal engram research or studies examining DG function through regional manipulations without ensemble‐specific targeting. Most of the studies are on fear engrams; where both fear and reward engrams are studied, this is indicated in the far left column. Within these criteria, Table 1 aims to comprehensively catalog DG engram studies through January 2026; the narrative text discusses representative studies from each theme rather than exhaustively covering all entries.

Abbreviations: GC, granule cell; LA, lateral amygdala; PFC, prefrontal cortex; SST, somatostatin.

This methodological trajectory has been tremendously productive, revealing fundamental principles of memory allocation, consolidation, retrieval, and circuit organization for fear engrams. Yet the focus on fear conditioning, while scientifically justified, has left critical questions largely unexplored: how does the DG process reward‐associated contextual memories, and are the mechanisms governing drug‐associated reward engrams fundamentally similar to or distinct from those governing fear engrams? Survival depends on remembering and navigating to rewarding locations (Sosa and Giocomo 2021). While reward processing occurs across multiple brain regions including those supporting context memory and navigation, how the brain encodes, stores, and retrieves reward‐associated memories to guide adaptive behavior remains incompletely understood.

The disparity in knowledge between fear and drug‐associated reward engrams reflects multiple converging factors. While ‘fear engrams’ have relatively consistent operational definitions in the literature, the field has yet to establish a unified framework for conceptualizing “reward engrams.” Rather than proposing a novel definition, this review synthesizes existing evidence examining context memory associated with different reward types, including natural rewards (food, sugar, and social interaction) and drugs of abuse (cocaine, opioids, and amphetamines). Early successes with fear conditioning created a self‐reinforcing research trajectory. Meanwhile, reward processing was predominantly studied in structures conceptualized as “limbic” or “motivational,” including the nucleus accumbens, ventral tegmental area, and amygdala. In contrast, the dentate gyrus framed as a “cognitive,” “spatial,” “contextual” memory structure has not been studied as extensively for reward‐associated context memory processing. However, accumulating evidence demonstrates that the hippocampus plays critical roles in reward‐related behaviors, including navigating for reward (Sosa and Giocomo 2021; Tessereau et al. 2025), discriminating between reward‐associated contexts (Sato et al. 2020), and integrating spatial information with motivational state (Gauthier and Tank 2018; Issa et al. 2024). The DG is necessary for associating rewards with specific spatial locations, including discriminating between closely spaced rewarded locations within an environment. The DG contributes to associating rewards with specific spatial locations by providing a stable, global representation of the environment that serves as a scaffold for spatially precise memory encoding in downstream CA1–CA3 circuits (Hainmueller and Bartos 2018).

Beyond historical paradigm bias, understanding hippocampal reward representations requires consideration of reward's temporal complexity. While fear conditioning provides a discrete, identifiable moment (shock delivery) for engram tagging, reward learning unfolds across multiple phases including anticipation, approach, receipt, and post‐reward evaluation. Reward signals in dopaminergic neurons typically relate to reward receipt or reward‐predicting cues, providing clear temporal anchors for analysis. This temporal complexity, combined with historical focus on fear paradigms, has left critical knowledge gaps about drug‐associated reward engrams. Yet, the literature suggests that the hippocampus does robustly encode reward‐related information and integrates reward salience into spatial representations. The hippocampus prioritizes coding for aspects of experience that are salient or relevant to the animal's goals, and reward represents a consistently salient event. Hippocampal cells cluster near and over‐represent rewarded locations, remain active at reward sites even when relocated, and, when optogenetically activated, drive reward‐seeking actions, suggesting that reward anchors hippocampal activity across the entire environment to create a map for experience in reference to remembered rewards in parallel to a map for space (Sosa et al. 2025). While these findings demonstrate robust hippocampal engagement with reward information, critical mechanistic questions about reward engram formation and maintenance remain unanswered. It remains largely unknown if drug‐associated reward engrams form through mechanisms similar to fear engrams, how they persist over time, and if different types of rewards (drug vs. “natural” reward) engage fundamentally distinct hippocampal processes. Understanding drug‐associated reward engram mechanisms in the DG has direct therapeutic relevance for addiction, depression, and other disorders where pathological reward processing drives maladaptive behavior.

This review examines dentate gyrus engram research through four perspectives. First, we discuss the DG's unique properties that make it ideal for engram studies. Second, we review seminal discoveries from fear engram research that established fundamental principles of memory allocation, consolidation, and retrieval. Third, we examine the emerging drug‐associated reward engram literature with particular attention to distinctions between drug‐associated and natural reward‐associated memories. Fourth, we consider comparative evidence that the DG can encode experiences of opposing valences. Together, these perspectives converge on a central observation: the DG exhibits remarkable plasticity in linking contextual representations to opposing emotional valences, a flexibility not found in brain regions with fixed valence‐coding populations, positioning it as a particularly promising target for modifying maladaptive emotional associations. Systematically investigating drug‐associated reward engrams with the same rigor applied to fear engrams is essential for determining whether the DG processes different valences through common principles or employs valence‐specific mechanisms, knowledge critical for advancing both basic engram neurobiology and translational applications for memory‐related psychiatric disorders.

## 2 The Dentate Gyrus: Anatomical and Functional Foundations for Engram Research

Four properties explain why the DG became the central model for fear engram research: circuit architecture and connectivity, sparse coding and pattern separation, adult neurogenesis, and dorsoventral functional specialization. These properties, combined with the methodological advantages of fear conditioning, made the DG exceptionally tractable for studying engrams for aversive memory.

### 2.1 Circuit Architecture and Connectivity

The hippocampus serves as a hub for declarative memory formation, playing a role in creating, storing, and retrieving episodic memories—a type of declarative memory that allows recall of events/experiences. A key feature of episodic memories is the dependence on contextual information. Not just the “what” but the “where” and “when” of a memory. Context refers to the sensory cues that are present in an environment/location during the time of encoding a memory. The hippocampus is essential for the formation and retrieval of contextual memories in mammals (Wiltgen et al. 2010). This structure consists of four main subregions: the DG, Cornu Ammonis 3 (CA3), Cornu Ammonis 2 (CA2), and Cornu Ammonis 1 (CA1), each with distinct cellular compositions, connectivity patterns, and computational functions (Borzello et al. 2023; Amaral et al. 2007) see (Farrell and Soltesz 2025) for alternative perspectives on hippocampal organization. Together, these regions form the trisynaptic circuit, a unidirectional flow of information that begins with inputs from the entorhinal cortex to the dentate gyrus, proceeds to CA3, and then to CA1, which serves as the primary output structure of the hippocampus back to the cortex (Amaral et al. 2007). Different regions of the hippocampus are implicated in different aspects of memory, with CA1 and DG most implicated in contextual and spatial learning and memory (Vasudevan et al. 2024).

The dentate gyrus occupies a “gating” position within the hippocampal memory system. The DG receives convergent input from different regions of the entorhinal cortex (EC), a six‐layered cortical structure and main source of excitatory input to the DG. The EC has two subdivisions, lateral (LEC) and medial (MEC), that carry distinct types of information. The LEC provides information about objects, odors, and other non‐spatial contextual features, while the MEC supplies predominantly spatial information through grid cells, border cells, speed and head direction cells (Hargreaves et al. 2007; Borzello et al. 2023; Hainmueller and Bartos 2020; Xu and Wilson 2012; Tsao et al. 2018; Kropff et al. 2015; Hardcastle et al. 2017). This convergence allows the DG to form conjunctive representations that bind spatial locations with the sensory features present at those locations (Knierim and Neunuebel 2016).

The principal neurons of the dentate gyrus are excitatory granule cells, which exhibit a unique physiology, comprise the vast majority of DG neurons, and serve as the primary computational units for processing incoming information (Borzello et al. 2023). Each granule cell forms powerful but sparse connections with CA3 pyramidal cells through large mossy fiber boutons that can drive large postsynaptic currents despite their small number (Henze et al. 2002), earning these synapses the moniker “detonator synapses.” This arrangement ensures that sparse activity in the DG can effectively propagate to downstream circuits. Additionally, granule cells form extensive collaterals that target hilar mossy cells and inhibitory interneurons, creating a complex local circuit that refines information processing through feed‐forward and feedback inhibition (Scharfman and Myers 2012).

### 2.2 Sparse Coding and Pattern Separation

The DG contains approximately four to five times more neurons than its input source, entorhinal cortex, or output target, CA3 (Poo et al. 2016). Local inhibitory circuits, primarily through GABAergic basket cells and hilar interneurons, impose tight control over granule cell activity (Amaral et al. 2007). This robust inhibition ensures that despite the large population of granule cells, only a sparse population (typically 2%–4%) responds to any given experience (Chawla et al. 2005; Leutgeb et al. 2007). This sparse coding in the DG is proposed to serve a computational function: pattern separation (Rolls 1996). Through this process, the DG transforms similar input patterns from the entorhinal cortex into distinct, non‐overlapping representations that can be differentially stored without interference. The DG also contains parvalbumin inhibitory neurons, the major inhibitory cell type of this region, that contribute to the sparsity of DG granule cell activity (Bartos et al. 2007; Lee et al. 2016). This sparse activation pattern of the DG granule cells allows for precise targeting of specific memory engrams without affecting unrelated memories.

At the cellular level, pattern separation emerges from the unique biophysical properties of dentate granule cells, which exhibit high input resistance, hyperpolarized resting membrane potentials, and strong dendritic filtering that makes them difficult to activate unless receiving precisely timed, convergent inputs (Schmidt‐Hieber et al. 2007). This cellular selectivity ensures that only specific input patterns can trigger granule cell firing, contributing to the formation of unique neural representations for even subtly different experiences (Yun et al. 2023).

### 2.3 Adult Neurogenesis and Circuit Plasticity

Although the total number of new cells in the mammalian brain depends on age and species (Amrein et al. 2011), the DG's continued generation of new neurons throughout adulthood (Altman and Das 1965) provides an additional layer of circuit plasticity not found in most other brain regions. Continuous generation of new neurons in the adult dentate gyrus fundamentally shapes how memories are encoded, maintained, and transformed over time (Deng et al. 2010), influencing both the formation of new engrams and the long‐term fate of existing memory traces.

Newborn granule cells (GCs) undergo a critical period of heightened excitability and enhanced synaptic plasticity before integrating into the existing circuit (Ninkovic et al. 2007; Dieni et al. 2013). Adult‐born GCs can modify signal processing in the DG and are necessary to perform specific tasks requiring discrimination of very similar situations. For example, immature neurons help distinguish between similar contexts and update existing contextual representations with new information (Nakashiba et al. 2012; Clelland et al. 2009). Ablation of adult‐born neurons impairs discrimination between similar contexts, highlighting their specific contribution to pattern separation (Sahay et al. 2011).

The impact of neurogenesis on memory extends across development and into adulthood, shaping both memory accessibility and precision. Early in life, the rapid forgetting of early‐life memories, termed infantile amnesia, coincides with peak rates of hippocampal neurogenesis. During this developmental window, newly generated neurons integrate into existing circuits and extensively remodel hippocampal connectivity. Causal experiments demonstrate that elevated neurogenesis directly drives accelerated forgetting during infancy (Akers et al. 2012). However, these memories are not permanently erased. Optogenetic reactivation of DG neurons that were active during initial memory encoding can recover seemingly lost infant memories in adulthood (Guskjolen et al. 2018), revealing that infantile amnesia reflects retrieval failure rather than storage failure. This memory recovery is associated with broader reactivation of tagged neuronal ensembles beyond the dentate gyrus, particularly in CA3, CA1, and cortical regions, positioning the DG as a critical access point to distributed memory networks.

In adulthood, neurogenesis continues to actively transform the resolution and precision of existing memory traces during systems consolidation. The DG initially stores detailed, specific memories that allow animals to discriminate between similar contexts, but neurogenesis gradually transforms these into more generalized representations. Cortical engrams, by contrast, encode generalized, low‐resolution representations at both recent and remote timepoints. When neurogenesis is eliminated, DG engrams remain in their original high‐resolution state (Golbabaei et al. 2025). This progressive transformation reflects neurogenesis‐dependent reorganization of hippocampal engram circuitry across the DG‐CA3‐CA1 axis, wherein newborn neurons reduce feed‐forward inhibition in CA3 and increase excitatory CA3‐to‐CA1 connectivity, enabling downstream engram neurons to become active beyond the original training context. Eliminating neurogenesis arrests this reorganization and preserves precise, context‐specific memories, while promoting neurogenesis accelerates the emergence of generalized ’gist’ memories (Ko et al. 2025), revealing that new neurons do not simply add storage capacity but actively remodel memory precision throughout life.

The relative contribution of young and mature granule cells to engram formation remains an active area of investigation. While young adult‐born neurons exhibit heightened excitability and enhanced synaptic plasticity that theoretically should favor their recruitment into engrams, activity‐dependent tagging studies indicate there is no preferential recruitment of young neurons relative to mature granule cells, suggesting that the transient plasticity of immature neurons does not translate into disproportionate engram participation (Denny et al. 2014). Instead, adult‐born granule cells appear to exert their primary influence on established engrams during post‐encoding consolidation, continuously integrating into existing hippocampal circuits and remodeling DG to CA3 synaptic connectivity in ways that transform precise episodic‐like memories into more generalized representations over time (Ko and Frankland 2021). This suggests that despite the enhanced plasticity and excitability of young neurons, mature granule cells, characterized by complex dendritic arbors, sparse activation, and established feedback inhibition, may be preferentially recruited into memory engrams.

The impact of neurogenesis on DG circuit function is further modulated by mossy cells, excitatory hilar neurons that exert both local and long‐distance control over granule cell activity (Scharfman and Myers 2012). Mossy cells regulate adult neurogenesis through direct synaptic connections onto neural progenitors and immature neurons as early as 5–14 days after birth, positioning them as gatekeepers of new neuron integration into existing circuits (Song et al. 2016). Beyond their role in neurogenesis, mossy cells may contribute to engram formation through their complex circuit mechanisms. Locally, mossy cells activate inhibitory interneurons that suppress granule cells within the same septotemporal domain, while their long‐range projections directly excite granule cells across the longitudinal axis of the hippocampus (Scharfman and Myers 2016). This dual mechanism may enable context‐specific engram allocation: local inhibition could sharpen pattern separation within a given context, while long‐range excitation could coordinate activity across distant DG regions. Selective manipulation of mossy cell activity during memory encoding could therefore alter which granule cells are recruited into engrams and how distinct engrams interact across hippocampal domains.

While the above evidence comes primarily from rodent studies, adult hippocampal neurogenesis also occurs in humans, though its extent and persistence remain debated (Seki 2020). Significant species differences, including variations in the molecular markers used to identify neurogenesis, highlight limitations of using classic models such as mice to fully recapitulate features of human adult hippocampal neurogenesis (Moreno‐Jiménez et al. 2021; Tosoni et al. 2023; Zhou et al. 2022, 2023). However, recent methodological advances combining immunohistochemistry, carbon‐14 dating, and single‐cell transcriptomics provide converging evidence for ongoing neurogenesis in the adult human dentate gyrus. Neurogenesis declines with age and in neurodegenerative conditions, suggesting potential therapeutic relevance. Importantly, functional properties of adult‐born neurons appear conserved across species, supporting the translational potential of neurogenesis‐based interventions derived from rodent engram studies. However, whether rodent engram findings translate to human therapeutic applications remains to be established.

### 2.4 Dorsoventral Functional Specialization

While the basic cytoarchitecture of hippocampal subfields is maintained along the dorsoventral axis, the dorsal and ventral hippocampus differ in many ways, such as activity patterns, gene expression, and connectivity. The dorsal hippocampus (DH) forms connections primarily with regions involved in spatial navigation and cognitive processing, including the retrosplenial cortex and anterior cingulate cortex. In contrast, the ventral hippocampus connects extensively with subcortical regions involved in stress responses, emotion, and motivation, such as the amygdala, hypothalamus, and nucleus accumbens. These anatomical differences support distinct functional roles, with the dorsal hippocampus primarily mediating spatial and contextual learning, while the ventral hippocampus regulates emotional and motivational aspects of behavior (Fanselow and Dong 2010).

These functional differences extend to the level of individual GC populations. Dorsal DG GCs are selectively required for contextual fear memory encoding, and hyperactivation of these cells impairs contextual learning and dramatically increases exploratory behavior in novel environments. In contrast, ventral DG GCs show no involvement in CFC, but their activation produces immediate anxiolytic effects without affecting overall locomotor activity (Kheirbek et al. 2013). Despite these distinctions, the DH and VH are not entirely functionally segregated and exhibit considerable functional overlap. For instance, the VH exhibits context‐dependent activity and coordinates the contextual retrieval of emotional memories (Jin and Maren 2015) and the DH contains ensembles that, when active, induce retrieval of emotional memories (Bernier et al. 2017; Chen et al. 2019; Garner et al. 2012; Josselyn and Tonegawa 2020; Ressler et al. 2021). This distributed yet interconnected organization is crucial for developing comprehensive therapeutic strategies that can address both the cognitive and emotional dimensions of memory‐related disorders. Recognizing this axis of specialization refines our understanding of how distinct hippocampal circuits may differentially contribute to symptom domains, even if targeted modulation remains a long‐term goal.

### 2.5 Molecular Tools for Engram Identification and Manipulation

The DG's experimental accessibility stems from the reliable expression of immediate‐early genes (IEGs) in neurons active during learning. IEGs such as c‐Fos, FosB, Arc, and Zif268 are rapidly and transiently expressed in neurons engaged during memory formation, producing a cellular “timestamp” that shows which neurons were active during specific experiences (Dragunow et al. 1992; Soulé et al. 2008). This breakthrough established IEGs as central to memory research, allowing investigators to not only identify but also manipulate memory‐bearing neurons with precision. IEGs are indispensable for long‐term memory, which itself depends on transcription and protein synthesis (Davis and Squire 1984; Dash et al. 1990; Silva et al. 1998; Kandel 2001; Asok et al. 2019). By serving as endogenous markers of neuronal activation, learning‐driven plasticity, and pharmacological modulation (Tregub et al. 2025; Hoffman et al. 1993; Guzowski et al. 1999; Barth et al. 2004; Okuno 2011; Minatohara et al. 2015; Salery et al. 2021), IEGs have become foundational tools for dissecting the molecular and cellular basis of memory.

Activity‐dependent tagging systems built on these foundations allow researchers to label neurons during learning experiences and later manipulate them with optogenetic or chemogenetic tools (Sakaguchi and Hayashi 2012; Lopez et al. 2024). These strategies typically use IEG promoter elements to drive expression of molecular effectors including: fluorescent reporters, optogenetic actuators, and chemogenetic receptors, in neurons activated during learning (Guenthner et al. 2013; Reijmers et al. 2007; Guzowski et al. 1999). Such tools, combined with modern imaging and circuit‐interrogation methods, have allowed researchers to label, monitor, and causally manipulate engram cells, providing direct evidence that these ensembles underlie the storage and retrieval of specific memories (Silva et al. 2009; Liu et al. 2012; Josselyn et al. 2015; Kandel et al. 2014; Josselyn and Tonegawa 2020; Reijmers et al. 2007; Kitamura et al. 2017; Tanaka and McHugh 2018; Tanaka et al. 2018; Vetere et al. 2019; Denny et al. 2014, 2017; Choi et al. 2018; Tonegawa, Pignatelli, et al. 2015; Yamamoto et al. 2021; Marks et al. 2022; de Ortega‐ San Luis and Ryan 2022; Terranova et al. 2022, 2023). Distinct IEGs contribute unique functions to these processes: c‐Fos is essential for consolidation and dendritic spine remodeling (Sagar et al. 1988; Fleischmann et al. 2003; Katche et al. 2010; Ryan et al. 2015; Choi et al. 2018; Yap and Greenberg 2018) but see (Uytiepo et al. 2025). Arc promotes memory persistence through synaptic plasticity (Plath et al. 2006; Minatohara et al. 2015; Guzowski et al. 1999; Okuno et al. 2012; Nikolaienko et al. 2018), and Npas4 regulates excitatory‐inhibitory balance needed for experience driven changes in synaptic plasticity to forge long term memory (Lin et al. 2008; Ramamoorthi et al. 2011; Spiegel et al. 2014; Sun and Lin 2016; Weng et al. 2018). Notably, IEG expression dynamics may reflect either co‐activation (Gonzales et al. 2020) or segregation across tasks and brain regions (Sun et al. 2020), highlighting their diverse roles in encoding experience.

The DG provides an ideal model system for IEG‐based engram identification. Granule cell activation produces robust, reliable IEG expression that distinguishes active from inactive populations. Repeated exposures to the same environment recruit overlapping DG ensembles, whereas distinct environments engage largely separate populations (Kubík et al. 2007; Satvat et al. 2011). These features make the DG uniquely suited for forming engrams that encode discrete contexts and experiences.

## 3 Fear Engrams in the Dentate Gyrus Foundational Discoveries and Emerging Principles

Building upon the unique properties of the DG, this section examines the key mechanistic discoveries gleaned primarily from fear conditioning research. Using activity‐based labeling techniques, extensive studies have described how engram cells contribute to various stages of memory formation and function including encoding, consolidation, reconsolidation, retrieval, and extinction. We organize these discoveries around eight themes to highlight that the field's most critical mechanistic principles are almost exclusively products of aversive memory studies. These discoveries are cataloged comprehensively in Table 1; the text below highlights representative studies that best illustrate each mechanistic principle.

### 3.1 Foundational Discoveries

Engram research began with the development of activity‐dependent tagging strategies that could precisely identify and manipulate memory‐bearing neurons. These technical advantages in tagging strategies converged in landmark studies that established the causal relationship between manipulation of DG activity and the recall and behavioral expression of memory. CFC became the paradigm of choice for these studies because it provides a quantifiable behavioral readout, freezing behavior, that directly reflects memory strength and recall.

The first direct evidence demonstrating the sufficiency of engram activity for memory recall came from a study in which DG granule cells active during CFC were labeled using a TetTag system (Reijmers et al. 2007) and equipped with channelrhodopsin‐2 (ChR2) for light‐controlled activation. Optogenetic reactivation of these tagged cells in a different neutral context elicited freezing behavior indicative of fear memory recall. Control experiments validated specificity: mice without prior shock training showed no freezing response despite light stimulation, and fear‐conditioned mice expressing only EYFP (a fluorescent marker without optogenetic function) also failed to show light‐induced freezing (Liu et al. 2012).

The modifiability of the engram was shown by creating false memories. Artificial activation of a neutral context engram *during* fear conditioning successfully linked the neutral context to the aversive shock outcome (Ramirez et al. 2013). This ability to swap contextual information within the engram demonstrates that DG memory traces contain modifiable content.

### 3.2 Engram Allocation and Competitive Dynamics

The mechanism of memory allocation, in which specific neurons become incorporated into an engram, is governed by neuronal excitability. This fundamental principle, first established in the lateral amygdala, dictates that neurons with higher intrinsic excitability are more likely to be incorporated into the engram (Han et al. 2009, 2007). Artificially increasing excitability creates a bias toward engram incorporation, while reducing excitability diminishes recruitment probability (Rashid et al. 2016; Sano et al. 2014; Zhou et al. 2009; Yiu et al. 2014). Artificially manipulating this excitability in the DG similarly biases engram allocation (Park et al. 2016).

Allocation in the DG is often described by a winner‐take‐all competitive process that ensures sparse coding. Active DG granule cells recruit somatostatin‐positive (SST+) interneurons, which then induce lateral inhibition of the surrounding, less‐active granule cells. This mechanism prevents neighboring neurons from joining the memory trace, which is critical for memory quality. An optimal engram size prevents interference while maintaining stability (Stefanelli et al. 2016).

The excitability of DG granule cells is actively regulated. Following memory recall, the rapid internalization of inward‐rectifier potassium channels Kir2.1 triggers a transient increase in engram cell excitability that lasts for about an hour. Artificially preventing this excitability enhancement by expressing exogenous Kir2.1 abolishes the recall‐induced enhancement of pattern separation and completion (Pignatelli et al. 2019). This suggests that intrinsic neuronal properties are not static but are dynamically modulated to control the circuit's plasticity and potentially bias future engram allocation.

While the competitive inhibitory model provides a strong framework for sparse coding, recent evidence suggests that engram allocation is highly context‐dependent and may not always rely on lateral inhibition. In one study, specific DG neural populations including semilunar granule cells are disproportionately incorporated into the engram due to their enhanced sustained firing and higher synaptic input frequency, without demonstrating an inhibitory effect on neighbors (Dovek et al. 2025). Ultimately, DG engram allocation appears to be governed by a dynamic interplay of intrinsic neuronal excitability, competitive microcircuits, and strong input‐driven synchronization, suggesting that the dominant mechanism varies across behavioral states.

### 3.3 Memory Consolidation and Silent Engrams

Memory consolidation transforms newly formed engrams into stable, long‐lasting traces through molecular, cellular, and systems‐level processes. However, consolidation does not always produce memories that are readily accessible. A critical discovery is that apparent memory loss often reflects retrieval impairment rather than engram destruction, with memories persisting in inaccessible “silent” engrams that can be recovered through targeted intervention. Despite amnesia during natural recall tests, direct optogenetic stimulation of original DG engram cells can restore robust freezing behavior, demonstrating that memory loss may reflect retrieval impairment rather than engram destruction (Ryan et al. 2015). Silent engrams retain their functional connectivity patterns but lack the synaptic strength to drive natural recall. Targeted synaptic potentiation can convert these silent traces to active engrams, providing mechanistic insight into memory recovery (Roy et al. 2017).

Sleep plays a regulatory role in determining whether engrams remain accessible. Sleep deprivation following memory encoding actively disrupts the necessary reactivation of DG engram neurons, and spatial transcriptomic profiling reveals that learning‐driven molecular signatures are fundamentally altered under sleep‐deprived conditions (Wang et al. 2024). Importantly, the structural integrity of these engrams remains intact despite behavioral retrieval failure. Object‐location memories formed under sleep‐deprived conditions can be recovered days later through either optogenetic activation of the original DG engram or pharmacological treatment with roflumilast, a clinically approved PDE4 inhibitor (Bolsius et al. 2023). These findings demonstrate that sleep deprivation creates suboptimal conditions for consolidation that render engrams inaccessible to natural retrieval cues without destroying the underlying memory trace.

At the molecular level, the stability of DG engrams depends on precise processes operating within critical time windows. CREB‐dependent transcriptional networks govern engram stability, including sustained expression of genes like Arc during the consolidation period (Rao‐Ruiz, Couey, et al. 2019). Epigenetic modifications such as increased methyltransferase activity within ensembles reinforce engram maintenance, strengthening long‐term memory retrieval fidelity (Gulmez Karaca et al. 2020). These molecular processes determine whether engrams consolidate into accessible or silent states.

Beyond molecular consolidation within the DG, memories undergo systems‐level reorganization over extended timescales. During systems consolidation, hippocampal engrams gradually become silent as the memory representation matures in the prefrontal cortex (Kitamura et al. 2017). This process involves coordinated changes across hippocampal subfields. Chronic two‐photon calcium imaging reveals the parallel emergence of stable and dynamic memory engrams across hippocampal subregions. CA1 and CA3 pyramidal neurons show precise, context‐specific but continuously changing representations, while DG granule cells maintain stable spatial codes over many days with low place‐ or context‐specificity (Hainmueller and Bartos 2018). This stability‐flexibility gradient across the hippocampal circuit may explain how memories can be both persistent and adaptable. Engrams dynamically evolve over time, refining their composition and circuit reliance. Inhibitory synaptic control and neuron dropout drive the transition for engrams to become selective (Tomé et al. 2024), suggesting that consolidation involves not just strengthening of relevant connections but also the active pruning of unnecessary circuit elements.

These findings collectively establish a framework for understanding dynamic accessibility of memory engrams. DG engrams exist along a continuum of accessibility states modulated by sleep, molecular consolidation processes, systems‐level reorganization, and environmental factors. Rather than functioning as a static storage system, the DG maintains a flexible engram network where apparent forgetting reflects altered retrieval dynamics rather than permanent memory loss. This framework has important implications: memories that appear lost may be recoverable through targeted reactivation, pharmacological intervention, or manipulation of retrieval conditions. The dynamic accessibility of DG engrams may enable adaptive regulation of which memories guide behavior based on their current relevance, while preserving traces that may become relevant in future contexts.

### 3.4 Memory Retrieval and Reactivation Dynamics

The ability of an engram to reactivate is a critical determinant of memory status. DG engram cells show consistent activation across contexts but recruit different downstream neural circuits based on environmental similarity. Brain‐wide mapping revealed that while DG engram cells themselves reactivate regardless of context, the broader network patterns they trigger vary depending on how closely the current environment matches the original encoding context. This dissociation between stable DG activation and flexible downstream recruitment suggests that reactivating DG engrams could be leveraged to override fear responses in triggering environments by redirecting downstream circuit engagement (Dorst et al. 2024).

Memory reactivation involves coordinated multi‐cellular architecture beyond neuronal activity alone. Neuronal and astrocytic activity exhibit synchronized calcium signatures during both natural and artificial memory recall, demonstrating that engram function depends on neuron–glia interactions rather than neuronal networks in isolation (Suthard et al. 2024; Dewa et al. 2025; Williamson et al. 2024). Engram reactivation patterns also predict memory performance during aging. The fidelity of engram reactivation serves as a biomarker for age‐related memory decline (Gulmez Karaca et al. 2021). Memory retrieval depends not only on which DG neurons reactivate but also on how they coordinate with glial cells and recruit downstream circuits. The flexibility in downstream recruitment despite stable DG reactivation provides a mechanistic framework for understanding how the same memory can be retrieved in different contexts and potentially be modified.

### 3.5 Fear Memory Processing: Competing Ensembles and Extinction Dynamics

The DG maintains competing fear engram populations that vie for behavioral control. Fear extinction recruits a distinct DG neuronal ensemble separate from the fear acquisition engram (Lacagnina et al. 2019). Using TetTag mice to label neurons active during fear conditioning versus extinction, separate DG ensembles were identified for each phase. Optogenetic activation of the extinction engram suppresses conditioned freezing, while activation of the fear acquisition engram reinstates freezing even in the absence of a fear cue. The relative activity between these acquisition and extinction engrams determines whether fear or safety behaviors predominate. This dynamic system supports ongoing memory updating and plasticity. Effective remote fear attenuation requires reactivation of the original fear engram (Khalaf et al. 2018). Enhanced DG engram activation during extinction training prevents subsequent fear renewal when animals are returned to the original conditioning context (Gong et al. 2022). The DG thus maintains competing engram populations, fear acquisition versus extinction, where relative ensemble activity determines behavioral expression, with lasting fear reduction dependent on establishing sufficiently strong extinction engrams that can suppress acquisition engrams across contexts and time.

### 3.6 Contextual Specificity: Mechanisms of Discrimination Versus Generalization

The DG is central to ensuring that memory retrieval remains contextually specific. The necessity of the DG in contextual precision is demonstrated by behavioral manipulations: DG inhibition during recall selectively impairs fear expression when subjects must distinguish between highly similar contexts, simultaneously increasing generalization and impairing extinction (Bernier et al. 2017).

Mechanistically, this specificity is governed by functional heterogeneity within the memory engram itself. Distinct neuronal populations, genetically defined by Fos‐ and Npas4‐dependent transcriptional pathways, regulate the discrimination‐generalization balance. The Fos‐dependent ensemble promotes memory generalization, receiving enhanced excitatory inputs from the medial entorhinal cortex, while the Npas4‐dependent ensemble promotes discrimination via enhanced inhibitory drive from local cholecystokinin‐expressing interneurons (Sun et al. 2020). Furthermore, molecular mechanisms tightly regulate these ensemble functions. The intrinsic excitability and autophagy protein expression within these ensembles determine the threshold for generalization, where downregulation of autophagy genes alters neuronal excitability and spine density, promoting generalization through differential effects on the targeted ensemble (Lin et al. 2025). These findings collectively establish that functional heterogeneity within memory engrams, regulated by multiple synaptic and molecular mechanisms, dictates specificity.

The precision of a memory is directly linked to the quality of its initial encoding. Experience‐dependent plasticity modulates specificity through engram size. Comprehensive memories acquired through extended learning generate larger engrams that support superior contextual discrimination, whereas impoverished memories from brief learning result in smaller engrams associated with behavioral overgeneralization (Leake et al. 2021).

Generalization reflects systematic reorganization of DG ensemble representations. Initially, contextual fear memories maintain specificity through preferential DG reactivation patterns that distinguish between contexts, but these distinctive patterns degrade as memories age, paralleling the behavioral transition from discrimination to generalization (Yokoyama and Matsuo 2016). At the circuit level, specific pathways control the expression of generalization. Activating the ventral DG (vDG) mossy cell‐dDG pathway selectively suppresses generalized, but not conditioned, fear, revealing a pathway for controlling memory specificity (Cui et al. 2024). Crucially, the long‐term maintenance of discrimination depends on activity‐dependent circuit modifications. Specifically, enhanced inhibitory connectivity between engram‐bearing granule cells and downstream CA3 interneurons (Guo et al. 2018).

The DG's regulatory capacity is sensitive to systemic disruption and external cues. Elevated glucocorticoids induce generalized contextual fear responses by selectively increasing DG granule cell excitability; this dysfunction can be prevented by chemogenetic suppression of the hyperactive DG cells (Lesuis et al. 2021). Ultimately, these competing ensemble dynamics, experience‐dependent plasticity, neuromodulatory sensitivity, and circuit‐level modifications determine whether memories remain contextually specific or become generalized.

### 3.7 Stress and Early Life Effects

The functional state of DG engrams is profoundly shaped by developmental experiences and ongoing environmental factors. Early‐life stress impairs the DG's capacity to encode robust, stable memories (Sanguino‐Gómez et al. 2024), while maternal immune activation paradoxically enhances engram ensemble size and dendritic spine plasticity, attenuating infantile amnesia (Power et al. 2023). Throughout the lifespan, DG engrams remain dynamically responsive to environmental context, with stressful social cues capable of reactivating fear engrams and potentiating aversive memory recall (Finkelstein et al. 2022).

### 3.8 Circuit‐Level and Network Interactions

The DG functions as a central hub within memory networks. Comprehensive brain‐wide mapping during contextual fear conditioning reveals coordinated activation across up to 117 distinct regions (Roy et al. 2022), many of which maintain functional connectivity with hippocampal networks. This hub‐like architecture is functionally significant. Simultaneous chemogenetic reactivation of multiple engram ensembles produces stronger recall than single‐region activation alone (Roy et al. 2022), demonstrating that distributed network coordination contributes to engram expression. Reward‐associated engrams similarly engage distributed networks including ventral hippocampus, nucleus accumbens, ventral tegmental area, amygdala, and prefrontal cortex, with the DG positioned at the entry point of hippocampal processing.

## 4 Reward Engrams Across Brain Regions and Circuits

The preceding sections document extensive progress in understanding fear engrams within the DG: their allocation mechanisms, consolidation dynamics, retrieval processes, and circuit‐level organization. Yet many of today's most pressing psychiatric conditions involve dysfunctional reward processing, including addiction, treatment‐resistant depression, anhedonia, and motivational deficits. This disparity between fear and reward engram research reflects both methodological and conceptual challenges.

At the methodological level, fear conditioning became the dominant framework for engram research, though not necessarily due to inherent experimental superiority. While freezing behavior provides a readily quantifiable readout, reward paradigms like conditioned place preference and self‐administration offer equally robust and well‐validated measures of learned associations. However, reward learning's temporal complexity unfolding across anticipation, approach, receipt, and evaluation phases requires additional experimental considerations for engram tagging that may have initially deterred systematic investigation. The prevalence of fear conditioning in engram research thus reflects both historical momentum and these added methodological considerations, rather than superiority of fear paradigms.

Conceptually, the clinical relevance of fear conditioning to anxiety disorders and PTSD generated sustained research momentum. The connection between basic reward learning mechanisms and complex addiction pathologies, by contrast, seemed less straightforward. Addiction involves compulsive drug‐seeking despite negative consequences, a phenomenon that extends beyond simple reward learning to encompass habit formation, craving, withdrawal, and decision‐making deficits. This apparent complexity may have deterred systematic investigation of fundamental reward engram mechanisms in favor of studying downstream addiction‐related processes.

**FIGURE 1:**
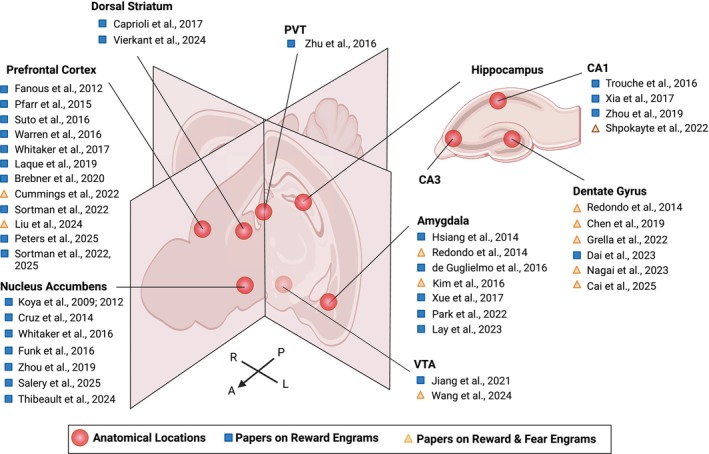
Schematic of mouse brain regions where reward memory engrams (both drug and natural reward) have been identified and manipulated, mapped onto a coronal and sagittal brain section. Red circles: Anatomical locations where reward engrams have been studied. Blue squares: Published papers on reward engrams. Gold triangles: Published papers where reward and fear engrams are studied in the same brain region. Brain regions indicated: Prefrontal cortex, dorsal striatum, nucleus accumbens, paraventricular nucleus of the thalamus (PVT), hippocampus (CA1, CA3, and dentate gyrus [DG]), amygdala, and ventral tegmental area (VTA). Anatomical axes: A, anterior; P, posterior; R, right lateral; L, left lateral. Adapted from (Tonegawa, Pignatelli, et al. 2015).

Despite these challenges, approximately 40 studies over the last twenty years have begun to characterize reward engrams (Figure 1), providing initial insights into appetitive memory encoding. Fear and reward engrams, though opposite in valence, converge on several common organizational principles. Both form through sparse allocation mechanisms that recruit only 2%–5% of neurons into functional ensembles (Mattson et al. 2008; Josselyn and Tonegawa 2020), both require CREB‐dependent transcriptional programs for stabilization (Park et al. 2016, 2022; Miyashita et al. 2018), and both preferentially engage cells with enhanced intrinsic excitability (Hsiang et al. 2014; Pignatelli et al. 2019). Moreover, similar methodological approaches including pharmacogenetic manipulation, IEG‐based activity tagging, and molecular genetic tools to selectively inhibit neuronal ensembles have been employed to study both fear and reward engrams.

While causal manipulations have established that reward engrams are necessary and sufficient for drug‐seeking behaviors (see Section 5), critical mechanistic questions remain unanswered. First, we lack comprehensive molecular characterization of reward engrams: what distinguishes drug‐associated engrams from natural reward engrams at the transcriptional, epigenetic, and proteomic levels? Fear engrams exhibit distinct molecular signatures including specific patterns of immediate‐early gene expression (c‐Fos, Arc, Npas4), CREB‐dependent transcriptional programs, and chromatin remodeling. Whether drug and natural reward engrams display similar molecular convergence or exhibit reward‐type‐specific molecular profiles remains largely unexplored. Second, the concept of “silent” engrams, neurons allocated to a memory trace that are necessary for retrieval but do not reactivate during recall, has been well characterized in fear circuits, where silent engram cells can be artificially reactivated to induce memory expression. Whether reward engrams similarly maintain silent populations, and whether these silent cells differ between drug and natural rewards, is unknown. Third, while fear extinction engrams have been extensively characterized as distinct neuronal populations that compete with acquisition engrams (Section 3.5), reward extinction engrams remain poorly understood. Do drug‐associated memories recruit extinction ensembles that suppress seeking behavior? Are these extinction ensembles stable over abstinence periods, or do they degrade during protracted withdrawal, contributing to relapse vulnerability? Fourth, the temporal dynamics of reward engram evolution across learning, consolidation, and reconsolidation phases require systematic investigation. Finally, potential sex differences in reward engram formation, stability, and reactivation, and the precise role of specific hippocampal subregions like the dentate gyrus in reward‐context encoding are largely unexplored. The following sections synthesize current knowledge of reward engrams while highlighting these critical mechanistic gaps.

### 4.1 Foundational Reward Engram Discoveries

A defining feature of addiction is the development of powerful, long‐lasting memories linking environmental contexts with drug effects, positioning addiction as fundamentally a disorder of learning and memory (Whitaker and Hope 2018; Goodman and Packard 2016). Causal manipulations of c‐Fos‐expressing ensembles have established their necessity for drug‐related learned behaviors across multiple brain regions, including the hippocampus, prefrontal cortex, nucleus accumbens, striatum, and amygdala, and across diverse drugs including heroin, cocaine, methamphetamine, and alcohol (Whitaker and Hope 2018; Goodman and Packard 2016). The specific contributions of each region to encoding drug‐related information and associations remain to be fully characterized.

The first drug‐associated reward engram study showed that selective inactivation of NAc ensemble populations disrupted context‐specific cocaine‐seeking (Koya et al. 2009). These studies employed conditioned place preference (CPP), a widely used paradigm for assessing drug‐context associations in rodents. In CPP, animals repeatedly receive drug administration in a specific location within an environment. When subsequently placed back into that environment without drug present, animals preferentially occupy the drug‐paired location, with time spent in each compartment quantifying the strength of the context‐drug association. Subsequent NAc studies (Koya et al. 2012; Cruz et al. 2015, 2014; Whitaker and Hope 2018; Whitaker et al. 2016; Zhu et al. 2016; Salery et al. 2025; Thibeault et al. 2024; Funk et al. 2016) demonstrated that drug‐associated reward engrams, like fear engrams, are stored in sparse, distributed populations that are both necessary and sufficient for memory‐guided behavior.

Cortical circuits show similar principles (Bossert et al. 2011; Cummings et al. 2022; Pfarr et al. 2015; Sortman et al. 2022, 2025; Whitaker et al. 2017; Suto et al. 2016; Laque et al. 2019). vmPFC neurons encode drug and natural reward memories through distinct ensembles (Warren et al. 2016, 2019; Bossert et al. 2011). dmPFC reward ensembles contain subpopulations encoding cues, reward delivery, and behavioral responses that emerge sequentially during learning (Grant et al. 2021), recruited from hyperexcitable neurons requiring regulated excitability for normal formation (Brebner et al. 2020). Cocaine and fear ensembles in dmPFC are functionally segregated: inhibiting cocaine‐tagged neurons suppresses cocaine seeking without affecting fear recall, and vice versa (Liu et al. 2024). Infralimbic cortex controls discriminative stimulus‐driven cocaine seeking after abstinence, while prelimbic cortex does not (Madangopal et al. 2021). In the orbitofrontal cortex (OFC), inactivating cue‐activated neurons reduced heroin seeking after prolonged but not short withdrawal, demonstrating that reward engrams undergo dynamic reorganization during abstinence (Fanous et al. 2012).

Amygdala studies established early allocation, consolidation, and retrieval principles (de Guglielmo et al. 2016; Hsiang et al. 2014; Zhou et al. 2009; Lay et al. 2023; Xue et al. 2017; Park et al. 2022). Neurons with elevated CREB are preferentially recruited, operating similarly for fear and reward (Zhou et al. 2009; Hsiang et al. 2014). Ensemble inactivation reversed alcohol dependence behaviors (de Guglielmo et al. 2016), while artificial reactivation induced reinstatement‐like behavior despite extinction (Park et al. 2022).

Dorsomedial striatum (DMS) ensembles play similarly critical roles: selective Daun02 inactivation of DMS neurons activated during methamphetamine‐associated cue exposure suppressed methamphetamine seeking after voluntary abstinence without affecting food seeking, demonstrating reward‐specificity of striatal ensembles (Caprioli et al. 2017). Extending these findings with higher temporal resolution, tagging of DMS neurons active specifically during alcohol consumption revealed that their optogenetic inhibition suppressed both alcohol seeking and taking, facilitated extinction, and reduced reinstatement of alcohol seeking (Vierkant et al. 2024).

These studies established technical and conceptual frameworks for reward engrams across brain regions; see (Cutler et al. 2025) for comprehensive review. The following sections examine how recent investigations have begun to extend reward engram research into hippocampal circuits, revealing both convergent principles and unique properties of DG reward encoding.

### 4.2 Drug‐Associated Engrams Versus Natural‐Reward Associated Engrams

Understanding whether drugs and natural rewards engage the same or distinct neuronal ensembles is crucial for targeting drug‐related reward engrams specifically. Drug‐associated memories could represent quantitative extremes of normal reward processing or fundamentally different encoding mechanisms. Many brain regions are activated by both reward types, but drug‐specific neuroadaptations render circuits like nucleus accumbens core, prelimbic cortex, and ventral tegmental area selectively necessary for drug‐seeking. However, most studies use between‐subjects designs comparing drug seeking in one cohort to natural reward seeking in another, with broad regional manipulations rather than engram‐level approaches (Nall et al. 2021). A key limitation noted in this review of the current literature is the scarcity of studies directly comparing drug and natural reward circuit necessity within the same subjects. Most investigations have employed between‐subjects designs, examining drug seeking (typically cocaine) in one cohort and natural reward seeking (typically sucrose) in another, using broad regional manipulations such as pharmacological inactivation or lesions rather than cell‐type‐specific or engram‐level approaches (Nall et al. 2021). Whether distinct DG ensembles encode different reward types or the same neurons encode both remains unknown.

The hippocampus presents a particularly complex picture. While heavily implicated in drug‐related behaviors including formation of drug‐stimulus associations, stress‐ and context‐induced relapse, and regulation of drug seeking via projections to the nucleus accumbens and prefrontal cortex (Goode and Maren 2019), manipulation studies reveal mixed evidence for drug selectivity. Deep brain stimulation of the hippocampus reduced reinstatement of both cocaine and sucrose seeking, suggesting shared necessity across reward types. However, suppressing hippocampal neurogenesis through irradiation increased cocaine self‐administration and context‐induced reinstatement in cocaine‐ but not sucrose‐seeking animals, indicating potential drug‐selective adaptations (Noonan et al. 2010). These mixed findings suggest that specific hippocampal subregions, projection pathways, or neuronal populations may differentially regulate drug versus natural reward seeking. To our knowledge, no studies have systemically investigated drug vs. natural context‐reward ensembles in the DG.

Activity‐dependent labeling reveals largely non‐overlapping populations for drug and natural rewards in other regions. Cocaine and sucrose ensembles in NAc core overlap only ~30% (Bobadilla et al. 2020). Cocaine ensemble reactivation during cued reinstatement correlates positively with seeking behavior, a relationship not observed for sucrose ensembles, indicating that drug‐associated ensembles may be more tightly linked to motivated behavior than those encoding natural rewards (Bobadilla et al. 2020). Extending beyond the NAcore, methamphetamine‐associated memories produce robust hippocampal engram reactivation in the DG, with methamphetamine‐encoding ensembles exhibiting elevated c‐Fos overlap and increased spine density during retrieval effects not observed for natural reward memories, suggesting that drugs of abuse may create contexts that are retrieved more readily than memories for natural rewards (Cai et al. 2025).

Drugs and natural rewards produce fundamentally different dorsal CA1 representations despite equivalent conditioned place preference scores (Sun and Giocomo 2022). During methamphetamine and morphine conditioning, spatial representations became orthogonalized with minimal overlap between contexts, contrasting with overlapping, stable representations during sucrose conditioning. This may reflect hyper‐strengthened indexing at the expense of configurational processing, making drug contexts compulsively retrieved while compromising spatial flexibility. These findings suggest the hippocampus does not simply encode drug contexts as ‘highly salient.’ Rather, drugs appear to fundamentally reorganize how spatial information is processed. Whether DG exhibits similar reorganization or maintains similar encoding across reward types is unknown. Given DG's role in pattern separation and position as hippocampal entry point, understanding differential processing of drug versus natural reward contexts is essential.

Individual differences complicate this picture. Prelimbic cortex shows stable representations within individuals, but some mice display high similarity for food and cocaine seeking while others show no overlap (Glanzberg et al. 2024). Prelimbic and anterior cingulate ensembles tagged during sucrose seeking show context‐dependent reactivation 2 weeks later, reduced in the same context but elevated in distinct contexts, with sex‐specific patterns (Jessen et al. 2022). Whether DG shows similar individual variability and context‐dependent reactivation is unknown.

Mechanistically, drugs of abuse may alter hippocampal function through at least two non‐mutually exclusive pathways. First, drugs may create abnormally strong or persistent engram ensembles. The enhanced stability of drug‐associated ensembles compared to natural reward ensembles supports this possibility. Enhanced indexing of drug contexts could lead to these memories dominating retrieval and behavior. Recent evidence suggests drugs hijack mesolimbic pathways that normally process homeostatic need states, recruiting these circuits for pathological drug‐seeking. This hijacking may explain why drug‐associated memories acquire abnormal persistence: drugs co‐opt neural mechanisms evolved to ensure survival‐critical behaviors, creating context‐drug associations that resist extinction similarly to associations linked to fundamental homeostatic drive (Tan et al. 2024). Second, drugs may disrupt the balance between hippocampal memory systems. Normal memory processing relies on balanced configurational processing (encoding specific spatial arrangements and features) and indexing (tagging and retrieving specific episodes or contexts). Drug‐associated memory may involve hyper‐strengthened indexing at the expense of configurational processing, as evidenced by the disPCp phenomenon where normal overlapping spatial representations are replaced by orthogonalized, context‐specific patterns (Sun and Giocomo 2022). The hijacking of homeostatic circuits may synergize with hippocampal reorganization: drugs activate mesolimbic pathways with unusual intensity while simultaneously distorting how the hippocampus encodes spatial contexts, creating a dual mechanism where both motivational drive and contextual memory become pathologically altered.

### 4.3 Hippocampal Reward Context Processing

To understand how these mechanisms specifically manifest in hippocampal circuits, recent studies have begun examining reward engrams across hippocampal subregions. Drugs of abuse produce powerful, persistent associations between the rewarding effects of drugs and the specific environments and cues present during those experiences (Crombag and Shaham 2002). Exposure to drug‐associated contexts increases c‐Fos expression in the hippocampus, as well as in the mPFC and NAc shell (Cruz et al. 2014), confirming hippocampal engagement during drug‐context retrieval. Several studies have explored whether manipulating hippocampal spatial representations can neutralize maladaptive drug associations, though primarily in CA1 manipulations.

CA1 hippocampal neurons play a role in encoding and retrieving drug‐reward contextual associations through distributed engram circuits. During nicotine and cocaine conditioning, specific CA1 neuronal ensembles emerge that encode drug‐reward contextual memories and are necessary for place preference expression (Xia et al. 2017). The ventral CA1 projects preferentially to the nucleus accumbens core, forming an engram circuit wherein both vCA1 and NAc core ensembles are necessary for memory retrieval, though activation of NAc core engrams alone is sufficient to retrieve cocaine memories (Zhou et al. 2019). At the ensemble level, vCA1 neurons encode stimuli with immediate behavioral relevance, forming representations that integrate environmental context with internal motivational states including threat processing and reward‐related learning, where ensembles rapidly reorganize to represent cues predicting reward and maintain stable representations across extinction, reinstatement, and even reversal of outcome valence (van der Veldt et al. 2025). This functional organization positions vHPC as encoding not simply spatial locations but rather what stimuli mean to the animal, whether they signal threat, reward opportunity, or social salience, with projection‐defined output channels showing functional heterogeneity. Drug‐context associations involve systematic remapping of CA1 place cells, with specific populations reducing spatial tuning in non‐drug contexts while maintaining representation of drug‐paired environments, creating orthogonal neural representations that correlate with drug‐seeking behavior (Sun and Giocomo 2022). Silencing CA1 neurons active during cocaine‐place conditioning and forcing alternative neurons to encode the same spatial context eliminates preference for the cocaine‐paired compartment while preserving the ability to form new place preferences, demonstrating that the cocaine‐associated context retains familiarity but loses emotional salience (Trouche et al. 2016). Place fields often cluster near reward locations, resulting in an overrepresentation of those locations by the neural population, and running toward a known goal location induces place‐specific firing along paths to goals distinct from firing during random foraging (Aoki et al. 2019; Dupret et al. 2010). Importantly, a subpopulation of hippocampal neurons appears specialized for encoding reward locations independent of spatial context, suggesting that hippocampal reward signals can be dissociated from general place firing (Gauthier and Tank 2018). Together, these findings raise the possibility that selectively editing hippocampal spatial representations could neutralize maladaptive drug‐context associations by disengaging the original engram ensemble and substituting an alternative representation.

Targeted optogenetic inhibition of dorsal hippocampal CA3 (dCA3) reveals a temporal window for disrupting cocaine‐context memories during reconsolidation. When dCA3 is inhibited immediately after re‐exposure to a cocaine‐paired context, during the early reconsolidation window when memories become transiently labile, subsequent context‐induced cocaine‐seeking behavior is significantly reduced. In contrast, inhibition at later time points or without prior memory reactivation produces no effect, demonstrating that dCA3 activity is specifically required for restabilizing reactivated cocaine memories (Qi et al. 2022). The inhibition affects both excitatory pyramidal neurons and GABAergic interneurons, suggesting that recurrent microcircuits within dCA3, rather than a single cell type, support memory maintenance during this labile phase (Qi et al. 2022).

While CA1 and CA3 studies demonstrate the broader hippocampal network's involvement in drug‐context associations, accumulating evidence positions the DG as a node for both storing and modulating drug‐associated engrams. Supporting evidence from lesion studies showing that DG damage impairs both contextual fear conditioning and cocaine‐induced conditioned place preference establishes that the DG is necessary for forming contextual associations with both aversive and appetitive stimuli (Hernández‐Rabaza et al. 2008). While these studies predate modern engram techniques, they provide foundational evidence for the DG's unique role in contextual memory formation. Although we lack detailed characterization of how DG ensembles encode reward contexts, recent work identifies the DG as a promising target for weakening pathological drug‐associated memories.

Using CPP as a model of methamphetamine‐reward associations, repeated hippocampal oxytocin microinjections effectively prevented drug‐primed reinstatement through dual mechanisms: enhanced adult neurogenesis and suppression of drug memory engrams in the DG (Cai et al. 2025). Oxytocin robustly promoted proliferation, survival, and morphological maturation of newborn DG neurons, increasing dendritic complexity, length, and branching while reversing methamphetamine‐induced impairments. Pharmacological or genetic depletion of newborn neurons abolished oxytocin's anti‐relapse effects, establishing a causal relationship between neurogenesis and memory disruption. Activity‐dependent labeling identified methamphetamine‐associated engrams in the DG that reactivated specifically during drug‐primed reinstatement. Oxytocin suppressed both engram reactivation (c‐Fos expression in tagged cells) and dendritic spine density within these ensembles. Chemogenetic reactivation of methamphetamine engrams reversed oxytocin's protective effects, demonstrating that engram suppression is functionally necessary for preventing relapse. This neurogenesis‐based intervention showed specificity for drug memories and while oxytocin induced forgetting of recent hippocampus‐dependent memories (spatial learning, contextual fear), remote hippocampus‐independent memories remained intact.

Further, the locus coeruleus (LC) to DG circuit is necessary for recall of naloxone‐precipitated conditioned place aversion in morphine‐dependent mice, a model of pathological aversion memory (Dai et al. 2023). Using naloxone‐precipitated conditioned place aversion (CPA) in morphine‐dependent mice, this circuit was identified as necessary for memory retrieval: chemogenetic inhibition of LC to DG transmission blocked CPA expression and reduced DG c‐Fos activation, preventing discrimination between saline‐paired and naloxone‐paired contexts. LC and DG, but not CA1, showed robust activation during memory recall, establishing the DG's preferential involvement in contextual discrimination tasks such as place‐paired conditioning paradigms and pattern separation. Optogenetic activation of LC to DG projections immediately before a recall‐extinction session significantly enhanced extinction learning and reduced subsequent aversive behavior. This enhancement operates through a reconsolidation‐based mechanism: recall reactivates the memory engram, rendering it transiently labile during a reconsolidation window, and extinction training during this window promotes memory updating rather than formation of a competing extinction ensemble. The effectiveness of this recall‐extinction procedure depends on successfully activating the original memory trace, and LC to DG stimulation serves this function by increasing the ensemble size of DG engram cells that are reactivated during extinction. Using Fos‐TRAP to permanently label CPA acquisition cells and c‐Fos immunohistochemistry to identify extinction‐active cells, successful extinction was associated with increased co‐localization between these populations, demonstrating that neurons tagged during initial memory formation are reactivated and updated during extinction rather than being replaced by a distinct population. In vivo calcium imaging confirmed that LC to DG activation expands this reactivated ensemble. Importantly, neurons tagged during the recall stage rather than the acquisition stage showed greater influence on extinction efficacy, indicating that extinction requires reactivation of hippocampally stored information and supporting the view that initially acquired memory and extinction memory represent distinct engram types within the DG. These findings reveal that opioid withdrawal‐related engrams may reside in the DG and support a reconsolidation‐based extinction model where recall‐induced engram reactivation drives memory modification. However, this study examined extinction of drug‐associated aversion (withdrawal‐induced negative affect), not extinction of drug‐seeking behavior itself. Whether extinction of rewarding drug memories such as those supporting conditioned place preference for reward operates through similar reconsolidation‐based reactivation mechanisms or instead recruits competing extinction ensembles akin to fear extinction remains unknown. The DG may employ distinct extinction strategies for aversive versus appetitive drug‐associated memories and/or natural rewards, and systematic investigation of reward memory extinction in the DG is needed to resolve this question.

Complementary work using activity‐dependent labeling in the VTA revealed that morphine‐positive ensembles (neurons recruited by initial morphine exposure) preferentially project to NAc and mediate dopamine‐dependent positive reinforcement (Jiang et al. 2021). However, chronic morphine escalation enhances inhibitory inputs from CRH neurons in the central amygdala onto these positive ensembles, driving negative affective states during opiate withdrawal. Suppressing CRHR1 in morphine‐positive ensembles weakened this inhibitory input and alleviated withdrawal‐induced negative affect, demonstrating that neurons initially encoding reward become targets of stress‐related inputs that produce aversive states during withdrawal. These findings establish that neuromodulatory manipulation of reward and aversion circuits spanning hippocampus, VTA, and amygdala may facilitate extinction of drug‐associated memories.

### 4.4 Context Identity, Valence Encoding, and Memory Plasticity

Emotional memories encode the affective value, or valence, of experiences. Neutral cues acquire positive or negative valence when paired with rewarding or aversive unconditioned stimuli, enabling them to drive approach or avoidance behaviors (Namburi et al. 2016, 2015; Williams et al. 2022). The hippocampal DG demonstrates remarkable flexibility in encoding experiences across the emotional valence spectrum, but the mechanisms enabling this bidirectional capacity remain incompletely understood.

A fundamental question for understanding DG engram function is whether hippocampal neurons encode stimulus or context identity versus emotional valence itself. Recent evidence suggests the hippocampus encodes “what this stimulus is” rather than “whether this is good or bad,” with valence computation occurring in downstream structures (Biane et al. 2025; Mohanta and Tye 2025). This identity‐encoding framework fundamentally reinterprets how we understand DG contributions to emotional memory and has critical implications for therapeutic targeting.

Calcium imaging in ventral CA1 during exposure to various appetitive and aversive stimuli reveals that distinct stimuli of the same valence (e.g., sucrose vs. female urine, both appetitive) activate separable vCA1 ensembles, while representations of individual stimuli remain stable even when their valence is experimentally reversed through conditioning (Biane et al. 2025). These findings suggest vCA1 maintains stimulus‐specific representations that report stimulus identity rather than valence per se, with valence computation potentially occurring in downstream targets such as amygdala and nucleus accumbens that receive indexed contextual information from the hippocampus. This framework positions the hippocampus as providing a context “index” that downstream valence‐computing circuits use to generate appropriate motivational responses.

Whether these findings support identity encoding versus valence encoding in the DG remains debatable. When DG neurons active during fear conditioning are labeled and optogenetically reactivated during reward conditioning in a neutral context, mice subsequently prefer the original fear‐paired context. Conversely, when reward‐tagged DG neurons are reactivated during fear conditioning, mice avoid the original reward‐paired context (Redondo et al. 2014). Under a valence‐encoding interpretation, individual DG neurons switch their intrinsic valence preferences. Under an identity‐encoding interpretation, context representations encoded in the DG are reassociated with different outcomes: the DG ensemble continues to encode “this is context X” while the association “context X predicts aversive/appetitive outcome” is updated through modifications in how the DG engram activates downstream valence‐computing circuits (Ortega‐de San Luis et al. 2023). This mechanism distinguishes the structural engram (the stable tagged cells encoding context identity) from the functional engram (the changeable behavioral output determined by which downstream circuits are activated) (Kim and Cho 2020).

However, the relationship between context identity encoding and valence processing may be more complex than a simple dichotomy. Other studies using immediate‐early gene expression, which captures neural activity integrated over longer periods than calcium imaging, have identified valence‐encoding ensembles in ventral hippocampus (Shpokayte et al. 2022). Partially segregated neuronal populations processing appetitive versus aversive information displayed distinct molecular signatures, projection targets, and receptor profiles. vCA1 neurons encoding appetitive experiences exhibited over 1000 differentially expressed genes and hundreds of differentially methylated genomic regions compared to neurons encoding aversive experiences. Three separate vCA1 populations were identified: two encoding either appetitive or aversive valence, and a third, smaller subset responsive to both. These valence‐selective populations innervate BLA and NAc through anatomically and molecularly distinct projections. This projection‐specific organization suggests vCA1 neurons may transmit contextual information through multiple parallel channels that can be independently modified, with transcriptional plasticity within engram cells potentially enabling switching by modifying specific axonal projection outputs.

Whether these apparently conflicting findings reflect true mechanistic differences, distinct timescales of valence processing (seconds‐to‐minutes for calcium dynamics versus hours for IEG expression), or regional heterogeneity within the hippocampus (dorsal vs. ventral, DG vs. CA1) remains an important question. One possibility is that the hippocampus employs multiple mechanisms: stable context identity encoding that operates on fast timescales, alongside slower valence‐selective molecular modifications and projection‐specific plasticity that determine which downstream circuits are recruited when a context is retrieved. Resolving these apparent contradictions requires systematic comparison of DG ensembles across emotional contexts using matched methodologies, timescales, and anatomical precision.

The DG's sparse coding and pattern separation properties may prioritize orthogonalizing different contexts regardless of their emotional significance. Alternatively, the DG may contain distinct ensembles or projection patterns for appetitive versus aversive contexts, similar to vCA1. Immediate‐early‐gene profiling reveals that molecular‐defined ensemble structure varies by region. The DG exhibits high baseline co‐expression with less experience‐dependent modulation, while PFC and BLA show more dynamic recruitment of double‐ and triple‐positive cells after appetitive or aversive experiences (Arai et al. 2025). Comprehensive molecular characterization comparing DG ensembles tagged during appetitive versus aversive experiences is needed to resolve whether the DG encodes context identity uniformly or exhibits valence‐selective features.

The DG's capacity for context‐outcome reassociation (Redondo et al. 2014) distinguishes it from structures like the basolateral amygdala, which contains genetically distinct, spatially segregated populations where anterior BLA Rspo2+ neurons encode negative valence and posterior BLA Ppp1r1b + neurons encode positive valence (Kim et al. 2016). These hardwired BLA populations cannot be readily re‐associated with outcomes of opposite valence. The DG's reassociation capacity likely reflects its role as a flexible indexing system: the same context representation can be linked to different downstream valence‐computing circuits depending on learning history. When a DG ensemble encoding “context X” is reactivated, whether it drives approach or avoidance depends on which projection pathways to BLA, NAc, or other targets have been strengthened through learning. This projection‐specific plasticity, rather than changes in how individual DG neurons intrinsically encode valence, may explain the flexibility observed in valence‐switching experiments. Similarly, projection‐defined valence organization shows that BLA neurons projecting to nucleus accumbens preferentially encode reward, whereas those projecting to central amygdala preferentially encode aversion, recruiting distinct local microcircuits (Beyeler et al. 2018). The contrast between DG flexibility and BLA stability raises an important mechanistic question. Does DG valence switching reflect true changes in how individual neurons encode emotional value, or does it represent reassociation of stable context representations with different outcomes? Valence switching experiments may demonstrate that context representations can be linked to different downstream valence‐computing circuits. This interpretation would be consistent with the DG's proposed role as an indexing system that tags contexts while downstream structures compute motivational significance.

DG engram manipulation extends beyond memory modification to direct modulation of affective states. Optical reactivation of a competing positive memory is sufficient to update a fear memory at the ensemble level, attenuating maladaptive conditioned fear (Grella and Donaldson 2024; Grella et al. 2022). Optogenetic reactivation of DG cells active during positive experiences acutely suppresses depression‐like behaviors in chronically stressed mice, including passive coping responses and anhedonia, requiring glutamatergic activity in the hippocampus–BLA–NAc pathway (Ramirez et al. 2015). While acute optogenetic activation produces immediate behavioral improvements that do not persist beyond stimulation, chronic reactivation of positive memory engrams over 5 days produces enduring antidepressant effects that outlast stimulation and correlate with restored hippocampal neurogenesis. However, the therapeutic outcome of chronic engram reactivation may depend not only on which memory is reactivated but on which hippocampal axis is targeted: chronic reactivation of fear engrams in the dorsal DG suppresses context‐specific fear independently of the BLA, whereas the same protocol applied to ventral DG fear engrams enhances fear through a BLA‐dependent mechanism (Chen et al. 2019).

Importantly, reactivating neutral or negative memory engrams fails to produce these beneficial effects. The therapeutic outcome depends on which engram is reactivated, not simply whether the DG is activated. This valence‐specificity has a circuit basis: positive memory engrams engage downstream reward‐processing pathways, and optogenetic inhibition of BLA terminals in the NAc abolishes the antidepressant‐like effects of positive engram reactivation. Upstream inputs also contribute to this selectivity. Dorsal raphe serotonergic neurons preferentially reactivate DG ensembles that were originally associated with positive experiences, providing a mechanism by which neuromodulatory systems could bias retrieval toward beneficial memories (Nagai et al. 2023). Together, these findings suggest that DG engrams function as access points to distributed emotional networks: activating a positive engram recruits reward circuitry, while activating a negative engram would recruit threat‐related circuits.

Collectively, these studies reveal the DG's capacity for bidirectional plasticity in emotional memory, distinguishing it from the more rigid valence encoding in the amygdala. However, critical mechanistic questions remain. Whether the DG encodes context identity or exhibits valence‐selective organization has not been directly tested in dorsal DG using comparable methods. Comprehensive molecular characterization of DG ensembles tagged during appetitive versus aversive experiences is lacking. Understanding whether the DG encodes context identity or valence has direct mechanistic implications. If the DG primarily indexes contexts while downstream structures compute valence, memory interventions could target context‐outcome associations without broadly disrupting emotional processing. If the DG exhibits valence‐selective populations like those in vCA1, interventions might need to differentially target appetitive versus aversive circuits. Closing the fear‐reward research gap through systematic comparison of DG ensembles across emotional contexts is therefore essential for completing our mechanistic understanding of DG function in emotional memory.

## 5 Therapeutic Implications: Leveraging Valence Plasticity for Treatment

Engram dysfunction refers to maladaptive changes in how memories are encoded, stored, or retrieved that contribute to pathological behaviors. Pathological associations form when disease‐related events become paired with co‐occurring neutral stimuli. Through mechanisms analogous to classical conditioning, these neutral stimuli acquire the capacity to trigger pathological responses across diverse conditions including reflex epileptic seizures, chronic pain, major depressive disorder, PTSD, and addiction (Balmer et al. 2025). In addiction, this manifests as abnormally strong, persistent associations between drugs and the environmental contexts and cues present during drug use. In fear‐based disorders such as PTSD, similar dysfunction produces excessively strong or overgeneralized associations between environmental cues and threat, leading to persistent avoidance and hyperarousal (Balmer et al. 2025).

The findings reviewed above establish several features that position the DG as a potential therapeutic target: engrams can be precisely allocated, modified, and reactivated; memories that appear lost may remain accessible through targeted intervention; and the DG exhibits remarkable plasticity in linking contextual representations to emotional outcomes. Whether these properties can be harnessed for clinical benefit depends on resolving fundamental mechanistic questions about reward engram encoding, particularly drug‐associated reward engrams, while developing practical methods for safe, targeted intervention in human populations. Current preclinical evidence demonstrates proof‐of‐concept across multiple intervention modalities and disease models, though substantial challenges remain in clinical translation.

Memory dysfunction in neurodegenerative disease and aging appears to reflect altered engram accessibility rather than permanent trace erasure. The preservation of engram substrates even in disease states raises the possibility that the DG could serve as a therapeutic target for memory‐related disorders. Alzheimer's disease models demonstrate that optogenetic activation of DG engram cells restores memory recall despite behavioral amnesia (Perusini et al. 2017), and structural deficits within engrams can be reversed through targeted synaptic potentiation (Roy et al. 2017). These findings establish proof‐of‐concept that DG engrams remain accessible to experimental manipulation even in disease states, supporting the feasibility of engram‐targeted interventions.

Adult neurogenesis provides a distinct avenue for therapeutic intervention. The intersection of engram dysfunction and impaired adult neurogenesis further contributes to memory impairment by disrupting engram formation and maintenance (Lazarov et al. 2024). Repeated hippocampal oxytocin microinjections prevented drug‐primed reinstatement through dual mechanisms: enhanced adult neurogenesis and suppression of drug memory engrams in the DG (Cai et al. 2025). This neurogenesis‐based intervention showed selectivity, inducing forgetting of recent hippocampus‐dependent memories while sparing remote hippocampus‐independent memories, suggesting that promoting circuit reorganization through neurogenesis could disrupt maladaptive memories underlying relapse in preclinical models. Whether such approaches translate to clinical populations remains an open question, though intranasal oxytocin has shown some efficacy in clinical trials for substance use disorders. More broadly, abnormalities in adult neurogenesis may contribute to memory dysfunction across multiple psychiatric conditions. Impaired neurogenesis could underlie pattern separation deficits in anxiety disorders and PTSD, where overgeneralization causes similar cues to trigger the same maladaptive memories (Kheirbek et al. 2012). Similar pathology may occur in addiction, where drug‐related memories become disproportionately accessible compared to memories of negative consequences (Scharfman and Myers 2016).

Beyond optogenetic and molecular approaches, some pharmacological and dietary interventions have shown effects on DG engram function in preclinical models. Grape‐derived polyphenol treatment increases both memory recall and neuronal recruitment to memory engrams in the DG (Smith et al. 2018). The clinically approved PDE4 inhibitor roflumilast was found to help recover inaccessible memories in rodent models (Bolsius et al. 2023), and retigabine, another clinically approved compound, rescued engram‐specific memory deficits by targeting distinct molecular pathways within discrete dentate gyrus engram populations (Jin et al. 2025), suggesting that some FDA‐approved compounds may influence engram function, though clinical efficacy for memory disorders remains to be established.

The DG's capacity for bidirectional plasticity in emotional memory raises questions about whether this property could be harnessed therapeutically. Unlike brain regions where emotional associations appear more fixed (such as the hardwired valence encoding in BLA). Disrupting context‐outcome associations formed in the DG could prevent pathological contexts from triggering maladaptive behaviors without erasing the context representation itself. Reassociating contexts with new outcomes could modify which downstream circuits are activated when a context is encountered. Biasing retrieval toward adaptive associations could shift which of multiple context‐outcome memories dominates behavior, similar to how serotonergic systems bias positive memory retrieval (Nagai et al. 2023). Targeting projection‐specific pathways could selectively weaken circuits that drive maladaptive behaviors while preserving adaptive functions.

For fear‐based pathologies such as PTSD, traditional approaches aim to weaken fear responses through extinction or pharmacological dampening. Whether DG‐targeted approaches could transform traumatic memory associations rather than merely suppress them remains a theoretical possibility requiring substantial further investigation. For addiction, while traditional treatments focus on blocking reward pathways or creating aversive associations, the capacity to modify drug‐context associations represents an alternative approach. Studies showing successful neutralization of drug‐place preferences through hippocampal manipulation provide proof‐of‐concept in rodent models, though clinical translation faces significant challenges.

The DG's position as a structure processing both fear and reward memories suggests it could theoretically serve as a target for conditions involving both fear and reward dysregulation, such as trauma‐related addiction or comorbid depression and anxiety. The DG's sparse coding and pattern separation functions could enable targeting of specific pathological contextual memories while leaving related adaptive memories intact, though achieving this specificity in clinical practice would require substantial methodological advances.

Memory dysfunction across neurodegenerative disease, aging, and other conditions appears to reflect altered engram accessibility rather than permanent trace erasure in many cases. Multiple intervention modalities have shown effects in preclinical models: optogenetic reactivation, synaptic potentiation, molecular restoration, pharmacological enhancement, and dietary modulation. However, significant challenges remain in translating these findings to clinical applications. Current techniques for engram manipulation rely on invasive optogenetic and chemogenetic approaches that are not readily applicable to human therapy. Developing non‐invasive methods for targeting specific engram populations based on molecular signatures or activity patterns represents a major technical hurdle. Additionally, the specificity required to modify pathological memories without disrupting adaptive memory function has not been demonstrated in complex, naturalistic contexts. The long‐term safety and efficacy of interventions that fundamentally alter memory representations remain unknown.

Furthermore, critical questions about reward engram mechanisms must be answered before therapeutic design becomes feasible. Whether drug‐associated memories engage the same mechanisms as natural reward memories or represent qualitatively distinct processes determines what interventions might be effective. Whether the DG encodes context identity or valence influences what aspect of memory should be targeted. How reward engrams consolidate, persist, and update over time informs when interventions might be most effective. The findings reviewed here establish that DG engrams are accessible to experimental manipulation and that such manipulations can produce behavioral changes in preclinical models. Whether these principles can be harnessed for clinical benefit requires substantial additional research addressing both fundamental mechanisms of reward memory encoding and practical methods for targeted, safe, and effective intervention in human populations.

## 6 Future Directions and Conclusions

This review documented substantial progress in understanding fear engrams alongside an emerging drug‐associated reward engram literature in the dentate gyrus. The unique properties that made the DG ideal for fear research (sparse coding, pattern separation, neurogenesis) remain incompletely leveraged for understanding reward mechanisms. Fully understanding the DG's capacity to process both aversive and appetitive experiences requires investigating how reward engrams form, consolidate, and express with the same rigor applied to fear memories.

Previous reviews have comprehensively covered engram research origins, methodological advances, and fundamental mechanisms (Mayford 2014; Hübener and Bonhoeffer 2010; Eichenbaum 2016; Han et al. 2022; Tanaka and McHugh 2018; Robins 2023; Josselyn et al. 2015, 2017; Tonegawa, Pignatelli, et al. 2015; Josselyn and Tonegawa 2020; Bostancıklıoğlu 2020; Poo et al. 2016; Tonegawa, Liu, et al. 2015; Whitaker and Hope 2018; Langille and Gallistel 2020; Nambu et al. 2022; Tonegawa et al. 2018; Najenson 2021; Brown and Banks 2015; Josselyn 2010; Gebicke‐Haerter 2014; Ryan et al. 2021; Rao‐Ruiz, Yu, et al. 2019; Dudai 2012; Devan et al. 2018; Choi and Kaang 2022; Sweis et al. 2021; O'Sullivan and Ryan 2024; Choucry et al. 2024; Peregrim and O'Leary 2024; Zaki and Cai 2024; Guskjolen and Cembrowski 2023; de Ortega‐ San Luis and Ryan 2022; Miry et al. 2020; Brodt and Gais 2021; Tomé et al. 2024; Rao‐Ruiz et al. 2021; Jeanneteau and Coutellier 2022; Ryan and Frankland 2022; Gräff and Ramirez 2024; Mattioni et al. 2023; Koob 2021; Salery et al. 2021; Müller 2013; Renteria et al. 2016; Denny et al. 2017). In contrast, this review has tried to address a specific gap: the pronounced research imbalance between fear and reward engram studies within the dentate gyrus, and the questions that emerge from understanding how this structure encodes experiences across the emotional valence spectrum. Our analysis reveals that while the DG has become a cornerstone for engram research, the field has developed with substantial emphasis on fear conditioning paradigms. This emphasis, driven by methodological advantages and established clinical relevance to anxiety disorders, has created knowledge gaps limiting our understanding of how positive emotional experiences and drug‐associated memories are encoded and maintained in hippocampal circuits.

The convergence of evidence establishes several principles about DG engram function. DG engrams are precisely allocated through competitive mechanisms based on neuronal excitability. These engrams undergo dynamic consolidation involving molecular, cellular, and circuit‐level changes. Retrieval involves context‐dependent reactivation producing behavioral outputs that can be modified. The DG contains functionally distinct ensembles regulating memory generalization versus discrimination. DG engrams exhibit plasticity, including capacity for reassociating contexts with different outcomes. Engram function is modulated by adult neurogenesis, sleep, stress, and complex network interactions. While many of these principles appear to extend from fear to reward research, critical questions remain about whether reward engrams, and specifically drug‐associated reward engrams, operate through identical or distinct mechanisms.

A fundamental unresolved question is whether hippocampal neurons encode stimulus or context identity versus valence itself. Recent evidence from ventral CA1 suggests that hippocampal representations may prioritize “what this stimulus is” over “whether this is rewarding or aversive,” with valence computation occurring in downstream structures (Biane et al. 2025). However, other work demonstrates valence‐selective molecular signatures and projection patterns within vCA1 (Shpokayte et al. 2022). Whether these findings reflect different timescales of analysis, regional heterogeneity within hippocampus, or distinct aspects of emotional memory encoding remains unclear. Comprehensive molecular characterization comparing DG ensembles tagged during appetitive versus aversive experiences is lacking. Ventral CA1 engrams display distinct molecular signatures for positive versus negative experiences, with over 1000 differentially expressed genes and hundreds of differentially methylated genomic regions (Shpokayte et al. 2022). Whether dorsal DG exhibits similar valence‐selective molecular features or encodes all contexts uniformly regardless of outcome remains unknown. Understanding these molecular distinctions could reveal why certain memories resist modification and identify potential therapeutic targets. Resolving whether the dorsal DG encodes context identity or exhibits valence‐selective organization has direct implications for how therapeutic interventions should be designed.

A critical gap exists in understanding whether different types of rewards engage the same hippocampal mechanisms. Drug‐associated memories and natural reward memories produce fundamentally different hippocampal representations in dorsal CA1, with drugs creating orthogonalized, context‐specific silencing patterns that natural rewards do not produce (Sun and Giocomo 2022). Whether the DG similarly distinguishes between drug and natural reward contexts, and whether this distinction reflects quantitative differences in memory strength or qualitative differences in encoding mechanisms, determines whether addiction‐related memories require distinct therapeutic approaches from other reward‐based disorders.

Cell‐type specific features distinguishing fear from reward engrams at cellular and circuit levels require systematic investigation. This includes identifying differences in intrinsic excitability, synaptic plasticity mechanisms, downstream projection patterns, and molecular profiles. Do fear and reward engrams utilize the same allocation rules, or do different neuronal subtypes preferentially encode different experiences? How do projection‐specific pathways (to NAc, BLA, lateral septum) differentially contribute to fear versus reward memory expression? Answering these questions requires technologies that combine activity‐dependent tagging with molecular profiling and projection tracing.

Developing clinically viable methods for targeting specific engram populations represents a major translational challenge. Current techniques rely on invasive approaches not readily applicable to human therapy. New strategies based on engram allocation principles, molecular signatures, or non‐invasive neuromodulation are needed. The discovery that certain FDA‐approved compounds can influence engram function in preclinical models (Bolsius et al. 2023) suggests that pharmacological approaches targeting molecular pathways underlying engram stability or retrieval may offer more immediately translatable options, though clinical efficacy remains to be established.

The few studies examining both fear and reward engrams have revealed that the DG can reassociate contextual information with different emotional outcomes (Redondo et al. 2014), distinguishing it from structures with more rigid valence encoding. Understanding how the DG processes and modifies emotional associations across the full valence spectrum requires comprehensive investigation of reward engram formation, modification, and expression. Only by systematically comparing fear and reward engrams using equivalent methodologies, molecular profiling approaches, and behavioral paradigms can we determine which principles are universal and which are valence‐specific. If fear and reward engrams operate through fundamentally different mechanisms, interventions may need to be tailored accordingly. If they share core properties with valence‐specific modifications occurring at the projection or molecular level, unified therapeutic strategies targeting the DG could potentially address multiple memory‐related disorders.

The substantial progress in fear engram research provides a roadmap for the field. The roadmap includes established methodologies for engram identification, tagging, manipulation, and characterization that can be applied to rewarding contexts. By bridging the fear‐reward research gap through systematic investigation of drug‐associated reward engrams, the field can develop integrated approaches offering a more comprehensive understanding of how emotional memories shape behavior and how targeting these memories might address the spectrum of psychiatric disorders where maladaptive memory processing drives pathological outcomes.

## Author Contributions

**Lorianna M. Colón:** conceptualization, data curation, formal analysis, funding acquisition, methodology, resources, supervision, writing – original draft, writing – review and editing. **Oluwatoni A. Famuyide:** data curation, formal analysis, methodology, writing – review and editing. **Amelia J. Eisch:** conceptualization, data curation, formal analysis, funding acquisition, investigation, project administration, resources, supervision, writing – original draft, writing – review and editing.

## Funding

This work was supported by Burroughs Wellcome Fund, Postdoctoral Enrichment Program (PDEP). National Institute of Mental Health (MH117628). National Institutes of Mental Health (MH129970). National Institute on Drug Abuse (F32DA062486). National Institute of Neurological Disorders and Stroke (NS088555, NS126279).

## Conflicts of Interest

The authors declare no conflicts of interest.

## Data Availability

Data sharing is not applicable to this article because no new data were created or analyzed in this study.
